# Treatment and clinical outcomes among stage II–IVA nasopharyngeal carcinoma patients with undetectable pretreatment Epstein-Barr viral DNA

**DOI:** 10.3389/fmed.2026.1752053

**Published:** 2026-08-27

**Authors:** Ci-ming Sun, Zhen Xu, Cheng-hao Li, Jun-xiang Chen, Xin-yi Su, Jing-xia Song, Shuang-hong Song, Qing-qing Xu, Yan-Ling Wu

**Affiliations:** 1 State Key Laboratory of Oncology in South China, Guangdong Key Laboratory of Nasopharyngeal Carcinoma Diagnosis and Therapy, Guangdong Provincial Clinical Research Center for Cancer, Sun Yat-sen University Cancer Center, Guangzhou, Guangdong, China; 2 Department of Radiation Oncology, National Cancer Center/National Clinical Research Center for Cancer/Cancer Hospital and Shenzhen Hospital, Chinese Academy of Medical Sciences and Peking Union Medical College, Shenzhen, Guangdong, China

**Keywords:** adjuvant chemotherapy, clinical outcome, concurrent chemotherapy, induction chemotherapy, locoregionally advancednasopharyngeal carcinoma, undetectable pretreatment EBV-DNA

## Abstract

**Background:**

This study analyzed the efficacy of multiple combined chemotherapy modalities on the long-term survival outcomes in stage II–IVA nasopharyngeal carcinoma (NPC) patients with undetectable pretreatment Epstein-Barr viral DNA (pre-DNA).

**Patients and methods:**

We retrospectively analyzed 2,440 patients with stage II–IVA NPC with undetectable pre-DNA. Patients received any of the following treatment modalities: intensity-modulated radiotherapy (IMRT) alone; concurrent chemoradiotherapy (CCRT); induction chemotherapy (IC) + CCRT; IC + IMRT; or CCRT + adjuvant chemotherapy (AC) at Sun Yat-sen University Cancer Center between 2009 and 2015. Propensity score matching (PSM) was performed to balance variables. Matched patients in different treatment subgroups were analyzed by comparing survival outcomes.

**Results:**

In the PSM cohorts, no significant differences were observed in disease-free survival (DFS), overall survival (OS), distant metastasis-free survival (DMFS), or locoregional relapse-free survival (LRRFS) between patients treated with IMRT combined with multiple chemotherapy modalities and those with IMRT alone (all *P* > 0.05). The estimated 5-year DFS, OS, DMFS, and LRRFS rates were 85.6% vs. 87.3% (*P* = 0.33), 93.5% vs. 92.2% (*P* = 0.64), 94.4% vs. 93.7% (*P* = 0.96), and 92.0% vs. 94.0% (*P* = 0.16), respectively. Similarly, no significant differences in 5-year DFS, OS, DMFS, or LRRFS were observed across subgroups receiving IMRT with or without concurrent chemotherapy (CC), IC followed by IMRT with or without CC, or CCRT with or without IC. However, among patients receiving CCRT with or without AC, the 5-year DFS (96.0% vs. 78.3%, *P* = 0.004), OS (98.0% vs. 88.1%, *P* = 0.027), and LRRFS (97.9% vs. 87.9%, *P* = 0.045) rates were significantly higher in patients without AC, whereas no significant difference was observed in DMFS (96.0% vs. 86.1%, *P* = 0.078). No significant differences in the survival outcomes of T3–4 stage and N2–3 disease were identified.

**Conclusions:**

In patients with stage II–IVA NPC with undetectable pre-treatment EBV DNA, the addition of chemotherapy to IMRT was not associated with a statistically significant improvement in survival, regardless of whether they received induction, concurrent, or adjuvant chemotherapy. Further prospective validation is required.

## 1 Introduction

Nasopharyngeal carcinoma (NPC) is a common malignant tumor primarily distributed in southern China (1). Moreover, the mortality rate is about 30 – 40 per 100,000 individuals in Guangdong (2). The pre-treatment plasma Epstein-Barr (EBV) DNA (pre-DNA) load is associated with tumor load, and represents one of the most important biomarkers of NPC (3, 4). More than 97% of newly diagnosed patients exhibit detectable pre-DNA (5). Several studies have reported a poor prognosis in patients with detectable pre-DNA compared to those with undetectable levels (3, 6). Radiotherapy (RT) is a radical treatment associated with a good response and a 5-year overall survival rate (OS) higher than 90% in patients with stage I NPC (7). Intensity modulated radiotherapy (IMRT) has greatly improved the locoregional control, and distant metastasis is now the main mechanism of treatment failure in locoregionally advanced NPC (8). The addition of chemotherapy to RT was found to improve the disease-free survival (DFS) rates for stage II–IVA patients, including induction chemotherapy (IC), adjuvant chemotherapy (AC), and concurrent chemotherapy (CC) (9–11). Therefore, IC + CCRT, CCRT, and CCRT + AC are currently recommended as treatment for patients with stage II–IVA NPC according to the National Comprehensive Cancer Network (NCCN) guidelines (12). However, the current treatment guidelines that recommend RT combined with chemotherapy are based on clinical trials that have included a very high proportion (97%) of patients with detectable pre-DNA, such as NRG-HN001 (NCT02135042). Moreover, the addition of IC to CCRT has been shown to improve the survival rates of patients with a pre-DNA concentration (IQR: 626 – 33,400) higher than 0 (5). The addition of CC to IMRT improved the 3-year OS of patients with pre-DNA levels of > 1,460 copies/mL (13).

Limited studies have focused on the clinical characteristics and prognostic outcomes of NPC patients with undetectable pre-DNA. A previous retrospective study including 480 NPC patients reported that 12.9% of patients remained consistently EBV-DNA undetectable throughout treatment. Compared with EBV-DNA positive patients, this subgroup presented with earlier clinical stage and demonstrated significantly better DFS, distant metastasis-free survival (DMFS), and OS (14). In addition, a study from non-endemic regions also showed that negative pre-treatment EBV-DNA is a favorable prognostic factor in patients with locally advanced NPC, and persistently undetectable EBV-DNA status before and after treatment confers an independent positive prognostic value (15). Although accumulating evidence consistently indicates better outcomes in EBV-DNA negative patients (14–16), it remains unclear whether the treatment strategy of radiotherapy plus chemotherapy recommended by NCCN guidelines is also applicable to patients with stage II–IVA NPC with undetectable pre-DNA. Treatment strategies specifically tailored to undetectable pre-DNA populations have not been adequately explored. Therefore, dedicated investigations focusing on this clinically distinct subgroup are still warranted.

Based on these considerations, this retrospective study was conducted to investigate the clinical characteristics, failure patterns, and survival outcomes associated with the addition of IC, CC, or AC to IMRT in II–IVA NPC patients with undetectable pre-DNA, aiming to provide evidence to support individualized treatment decision-making for this specific patient population.

## 2 Materials and methods

### 2.1 Study subjects

**Figure 1:**
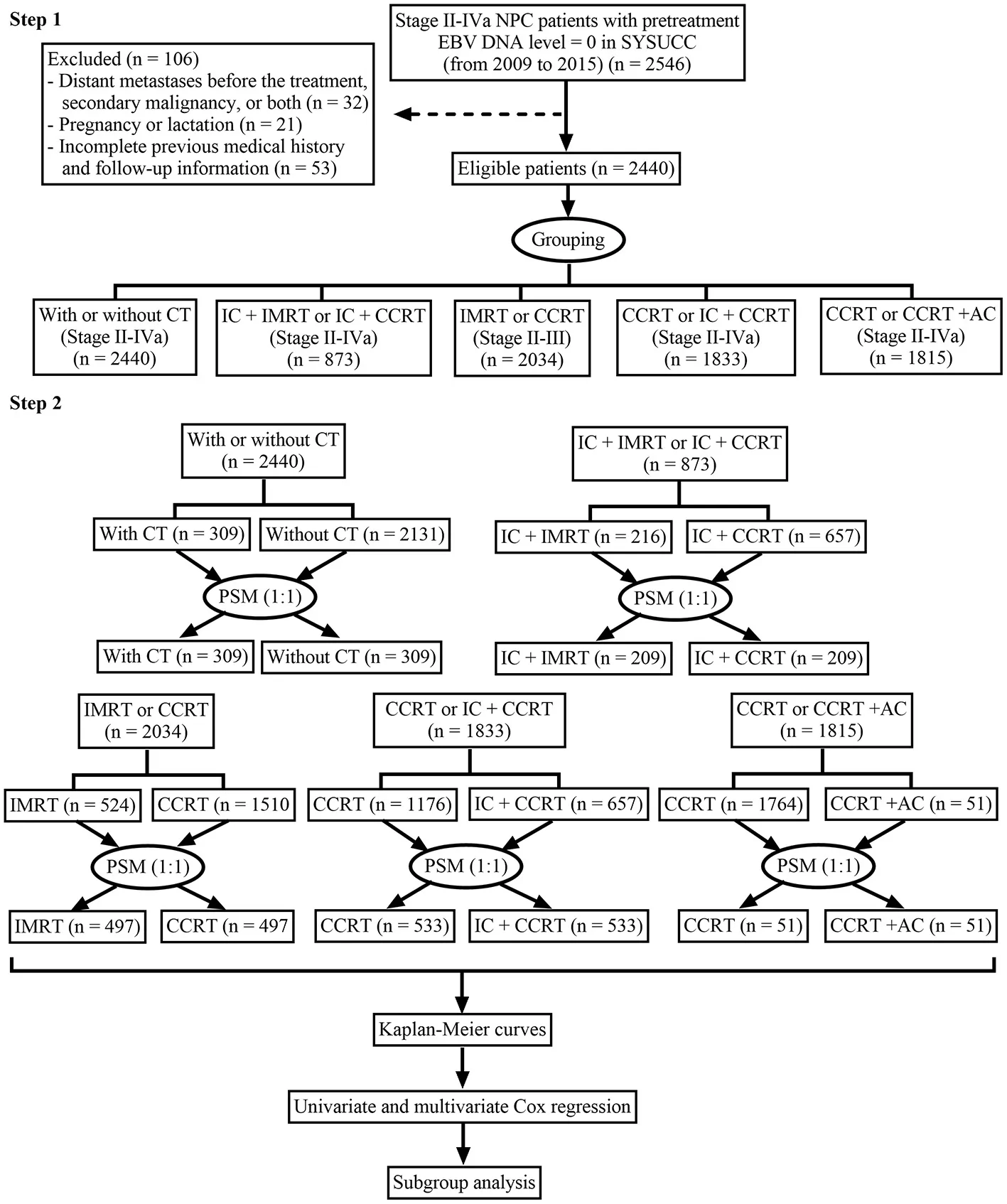
Flow chart of the study design. NPC, nasopharyngeal carcinoma; SYSUCC, Sun Yat-Sen University Cancer Center; IMRT, intensity-modulated radiotherapy; CT, chemotherapy; IC, induction chemotherapy; AC, adjuvant chemotherapy; CCRT, concurrent chemoradiotherapy; PSM, propensity score matching analysis.

A total of 2,546 patients with previously untreated, biopsy-confirmed NPC, restaged as II–IVA disease [according to the 8th edition American Joint Committee on Cancer (AJCC) staging system for NPC (17)], with undetectable pre-DNA, and treated at the Sun Yat-sen University Cancer Center (SYSUCC) from June 2009 to December 2015 were retrospectively included in this study. The following exclusion criteria were used: 1) evidence of distant metastasis prior to treatment, secondary malignancy, or both; 2) pregnant or lactating women; and 3) a lack of the necessary information. A flow chart describing the eligible patient criteria is shown in Figure 1. Finally, a total of 2,440 patients were included in this study. The ethics committee of SYSUCC approved our study protocol and the Institutional Review Board waived the requirement for patient written informed consent. The research protocol was formulated in accordance with the relevant requirements of the World Medical Association's Declaration of Helsinki.

### 2.2 Sampling and assay of plasma EBV DNA

Plasma EBV DNA was extracted using a QIAamp Blood Kit (Qiagen, Hilden, Germany), and DNA concentrations were routinely measured with real-time quantitative polymerase chain reaction (18), as described in the Supporting Materials. According to the testing kit adopted by our hospital, the sensitivity, including the lower limit of detection and the lower limit of quantification, is 400 copies/mL.

### 2.3 Chemotherapy and radiotherapy

All patients received intensity-modulated radiotherapy (IMRT). Delineation of the target volume was consistent with the treatment protocol at our institution (19) and the International Commission on Radiation Units and Measurement Reports 50 and 62 (20, 21). A total dose of 66–72 Gy at 2.12–2.27 Gy per fraction was prescribed to the planning target volume (PTV) of the primary gross tumor volume (GTVnx), while 64–70 Gy in 30–33 fractions was prescribed to the PTV of the involved lymph nodes (GTVnd). The PTVs of the high-risk and low-risk clinical target volumes (CTV1 and CTV2) received 60–63 Gy and 50–54 Gy, respectively, in 30–33 fractions.

In accordance with the NCCN guidelines, patients with stage II–IVA disease were recommended to receive IMRT with or without induction, concurrent, or adjuvant chemotherapy. Among the 2,440 patients included in this study, 2,131 (87.3%) received chemotherapy. For IC or AC, the main regimens included TP (docetaxel 75 mg/m^2^ on day 1 plus cisplatin 75 mg/m^2^ on day 1), PF (cisplatin 80 mg/m^2^ on day 1 combined with fluorouracil 800 mg/m^2^ per day as continuous intravenous infusion on days 1–5), and TPF (docetaxel 60 mg/m^2^ on day 1, cisplatin 60 mg/m^2^ on day 1, and fluorouracil 600 mg/m^2^ per day on days 1–5), administered every 3 weeks for 2–3 cycles. CC was mainly based on cisplatin administered on days 1, 22, and 43 during radiotherapy, with a typical dose of 80–100 mg/m^2^ every 3 weeks. The decision to omit chemotherapy was according to individual clinical judgment – taking into account physician experience that undetectable EBV DNA usually indicates low risk of disease failure.

### 2.4 Follow-up and endpoints

The endpoints of this study included DFS, defined as the time from treatment initiation to disease failure or death from any cause; OS, the time from treatment initiation to death from any cause or last follow-up; DMFS, the time from treatment initiation to remote distant metastasis or death from any cause; and locoregional relapse–free survival (LRRFS), the time from treatment initiation to locoregional relapse or death from any cause. Within the first 3 years of follow-up, the patients were assessed every 3 months, followed by an evaluation every 6 months thereafter until death. The median follow-up time was 69.8 months (range: 4.5 −123.0 months) for the whole cohort. Whenever feasible, local, regional, or distant relapse was confirmed by fine-needle aspiration or biopsy.

### 2.5 Statistical analysis

Propensity scores were calculated using a logistic regression analysis for each patient with the following covariates: age, gender, smoking, drinking, *T* stage, *N* stage, and overall stage. Patients were matched using a 1:1 nearest-neighbor matching algorithm with a caliper of 0.02 × standard deviation of the logit of the propensity score, without replacement. All subsequent survival analyses were performed on the matched cohort. The survival outcomes (time-to-event endpoints) of different treatment subsets (IMRT with or without chemotherapy, IMRT with or without CC, IC followed by IMRT with or without CC, CCRT with or without IC, CCRT with or without AC) were analyzed using Kaplan-Meier survival curves. A log-rank test was performed to assess differences between the groups. Univariate Cox regression analyses were performed for gender (female vs. male), age (≥45 vs. < 45 years), smoking, drinking, family history, tumor stage, nodal stage, and treatment modalities, and variables with *P* < 0.10 together with the treatment group were subsequently entered into the multivariable Cox model to identify independent prognostic factors. SPSS version 22.0 (IBM Corporation, Armonk, NY, USA) and R version 4.0.2 software (http://www.r-project.org) were used to calculate the statistical analyses. A *P* value < 0.05 was indicated as statistical significance. Summary statistics for time-to-event data were evaluated with hazard ratios (HRs) and 95% confidence intervals (CIs).

## 3 Results

### 3.1 Clinical outcomes of patients treated with IMRT with or without chemotherapy

**Figure 2:**
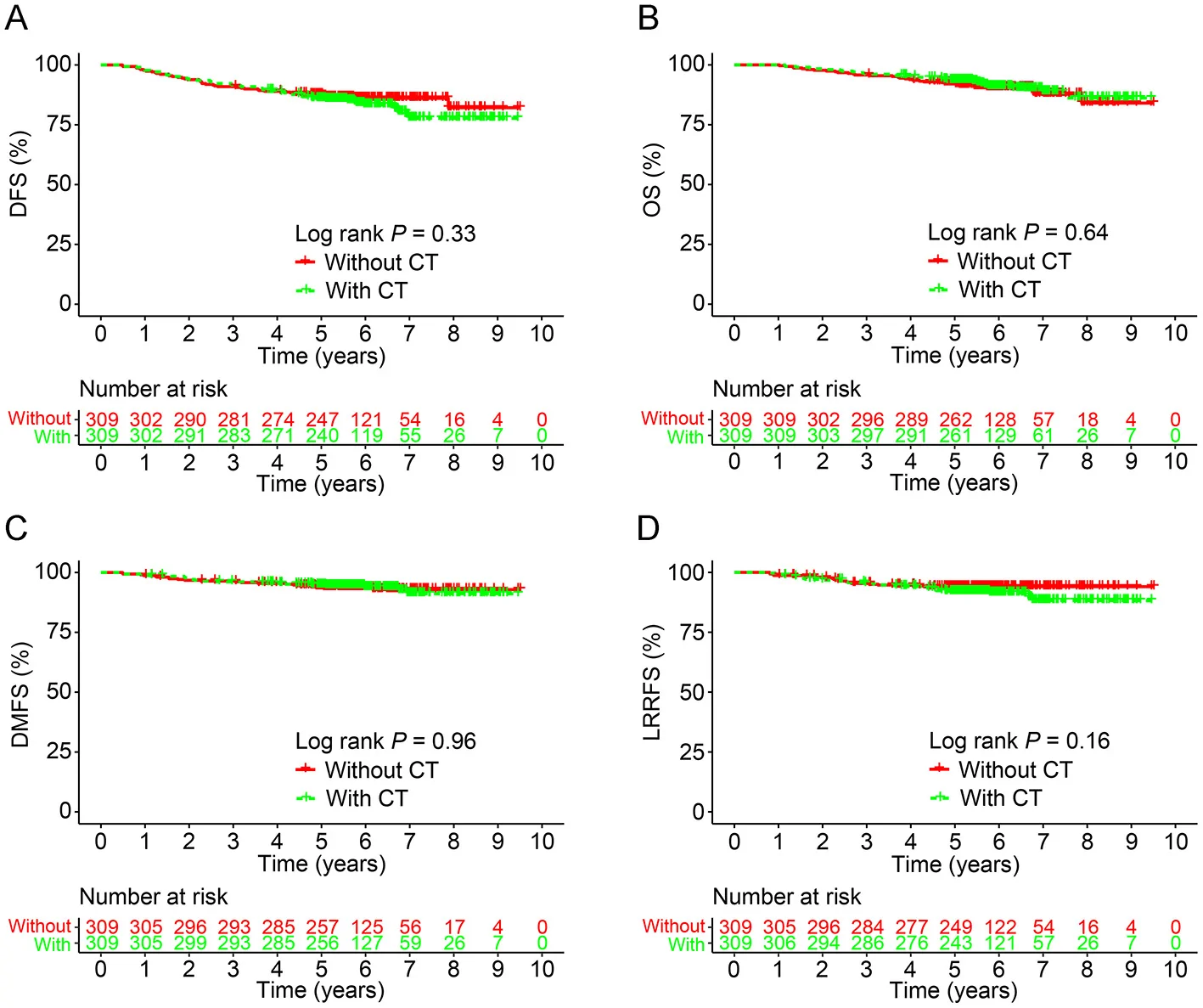
Kaplan-Meier curves for disease-free survival (DFS) **(A)**, overall survival (OS) **(B)**, distant metastasis-free survival (DMFS) **(C)**, and locoregional relapse-free survival (LRRFS) **(D)** of patients between the intensity-modulated radiotherapy + chemotherapy (IMRT + CT) vs IMRT alone groups.

**Figure 3:**
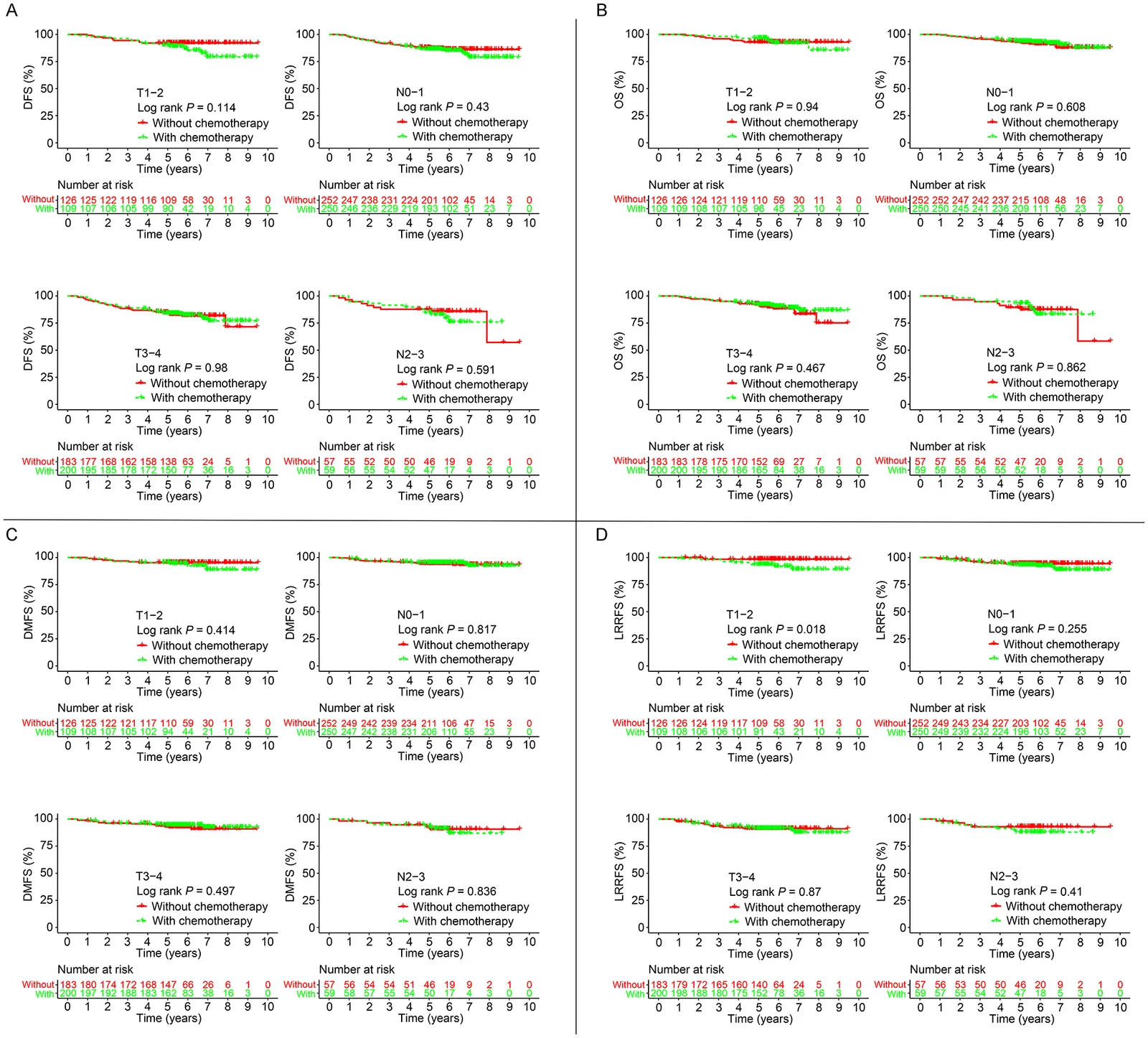
Subgroup analysis of patients based on different risk stratification (i.e., T1–2, T3–4, N0–1, and N2–3) between the IMRT + CT vs IMRT alone groups for disease-free survival (DFS) **(A)**, overall survival (OS) **(B)**, distant metastasis-free survival (DMFS) **(C)**, and locoregional relapse-free survival (LRRFS) **(D)**.

Among the 2,440 patients included in this study, the male-to-female ratio was 2.7:1 (1,775 vs. 665), with a median age of 45 years (range, 18–79). Following one-to-one propensity score matching, 309 matched pairs of patients (*n* = 618) treated with IMRT combined with chemotherapy vs. IMRT alone were identified. Baseline characteristics were well balanced between the two groups after matching, with all *P* values greater than 0.05 (Table 1). The 5-year DFS, OS, DMFS, and LRRFS rates for the 309 selected pairs were 86.7%, 92.8%, 94.2% and 93.0%, respectively. The estimated 5-year DFS, OS, DMFS, and LRRFS were 85.6 and 87.3% (*P* = 0.33), 93.5 and 92.2% (*P* = 0.64), 94.4 and 93.7% (*P* = 0.96), and 92.0 and 94.0% (*P* = 0.16), respectively, in patients with or without chemotherapy (Figures 2A–D). In multivariable analysis, smoking and N stage were independent prognostic indicators of OS, while the treatment group (IMRT with CT vs. IMRT alone) did not show a significant association (Table 2). Further subgroup analyses according to risk stratification (T1–2, T3–4, N0–1, and N2–3) showed no significant differences in 5-year DFS, OS, or DMFS and LRRFS between the two treatment groups, except for LRRFS in the T1–2 subgroup (Figures 3A–D).

**Table 1:** Clinical characteristics of nasopharyngeal carcinoma patients treated with IMRT with or without chemotherapy in the original and matched cohorts.

Characteristic	Original cohort		*P*-value	Matched cohort		*P*-value
	Without CT	With CT		Without CT	With CT	
	(*n* = 309)	(*n* = 2131)		(*n* = 309)	(*n* = 309)	
**Gender**			0.324			0.926
Female	77 (24.92%)	588 (27.59%)		77 (24.92%)	78 (25.24%)	
Male	232 (75.08%)	1543 (72.41%)		232 (75.08%)	231 (74.76%)	
**Age (years old)**			0.145			0.808
≥45	170 (55.02%)	1078 (50.59%)		170 (55.02%)	173 (55.99%)	
< 45	139 (44.98%)	1053 (49.41%)		139 (44.98%)	136 (44.01%)	
**Smoking**			0.442			0.668
Yes	98 (31.72%)	723 (33.93%)		98 (31.72%)	103 (33.33%)	
No	211 (68.28%)	1408 (66.07%)		211 (68.28%)	206 (66.67%)	
**Drinking**			0.635			0.481
Yes	39 (12.62%)	290 (13.61%)		39 (12.62%)	45 (14.56%)	
No	270 (87.38%)	1841 (86.39%)		270 (87.38%)	264 (85.44%)	
**Family history**			0.191			0.605
Yes	96 (31.07%)	586 (27.5%)		96 (31.07%)	102 (33.01%)	
No	213 (68.93%)	1545 (72.5%)		213 (68.93%)	207 (66.99%)	
**KPS**			0.977			0.865
≥90	291 (94.17%)	2006 (94.13%)		291 (94.17%)	290 (93.85%)	
< 90	18 (5.83%)	125 (5.87%)		18 (5.83%)	19 (6.15%)	
**8th T stage**			0.158			0.406
T1	47 (15.21%)	343 (16.1%)		47 (15.21%)	44 (14.24%)	
T2	79 (25.57%)	440 (20.65%)		79 (25.57%)	65 (21.04%)	
T3	139 (44.98%)	1074 (50.4%)		139 (44.98%)	159 (51.46%)	
T4	44 (14.24%)	274 (12.86%)		44 (14.24%)	41(13.27%)	
**8th N stage**			0.347			0.87
No	76 (24.6%)	455 (21.35%)		76 (24.6%)	79 (25.57%)	
N1	176 (56.96%)	1264 (59.31%)		176 (56.96%)	171 (55.34%)	
N2	40 (12.94%)	321 (15.06%)		40 (12.94%)	45 (14.56%)	
N3	17 (5.5%)	91 (4.27%)		17 (5.5%)	14 (4.53%)	

All continuous variables were changed to categorical variables. A Pearson χ^2^ test was used to calculate the *P*-value. IMRT, intensity-modulated radiotherapy; CT, chemotherapy.

**Table 2:** Univariable and multivariable analyses of prognostic factors for OS.

Characteristic	Univariable analyses		Multivariable analyses	
	HR (95% CI)	*P* value	HR (95% CI)	*P* value
**CT (Yes vs No)**	0.886 (0.531–1.476)	0.641	0.871 (0.522–1.454)	0.597
Gender (Female vs. Male)	0.615 (0.312–1.216)	0.162		
Age (>=45 vs. < 45)	1.703 (0.993–2.922)	0.053	1.610 (0.929–2.790)	0.090
Smoking (Yes vs. No)	1.942 (1.165–3.237)	**0.011**	1.762 (1.044–2.973)	**0.034**
Drinking (Yes vs. No)	1.698(0.901–3.201)	0.102		
Family history	0.648 (0.356–1.180)	0.156		
*T* stage (vs T1)	0.168			0.177
T2	0.281 (0.091–0.873)	**0.028**	0.285 (0.091–0.896)	**0.032**
T3	0.612 (0.279–1.341)	0.220	0.743 (0.337–1.640)	0.463
T4	0.700 (0.358–1.368)	0.297	0.849 (0.430–1.674)	0.636
*N* stage (vs No)	**0.037**			**0.021**
N1	0.204 (0.068–0.607)	**0.004**	0.181 (0.060–0.544)	**0.002**
N2	0.455 (0.191–1.081)	**0.074**	0.479 (0.199–1.155)	0.101
N3	0.537 (0.195–1.477)	0.228	0.509 (0.183–1.417)	0.196
**CCRT (vs IMRT)**	0.781 (0.493–1.238)	0.294	0.772 (0.487–1.224)	0.272
Gender (Female vs Male)	0.651 (0.374–1.132)	0.128		
Age (>=45 vs < 45)	1.845 (1.113–3.059)	**0.018**	1.779(1.069–2.962)	**0.027**
Smoking (Yes vs No)	1.773 (1.117–2.815)	**0.015**	1.719 (1.080–2.737)	**0.022**
Drinking (Yes vs No)	1.092(0.524–2.275)	0.814		
Family history	0.850 (0.513–1.410)	0.529		
T stage (vs T1)	0.471			
T2	0.688 (0.372–1.273)	0.234		
T3	0.973 (0.578–1.638)	0.918		
N stage (vs No)	0.073			0.050
N1	0.393 (0.175–0.886)	**0.024**	0.375 (0.166–0.845)	**0.018**
N2	0.643 (0.326–1.269)	0.203	0.658 (0.333–1.300)	0.228
**IC+CCRT (vs IC+** **IMRT)**	0.838 (0.486–1.446)	0.526	0.670 (0.379–1.183)	0.167
Gender (Female vs Male)	1.902 (1.044–3.465)	**0.036**		
Age (>=45 vs < 45)	0.657 (0.338–1.279)	0.216	1.781 (0.957–3.315)	0.069
Smoking (Yes vs No)	1.609 (0.928–2.790)	0.090	1.420 (0.807–2.499)	0.224
Drinking (Yes vs No)	1.376(0.588–3.222)	0.462		
Family history	1.268 (0.703–2.287)	0.429		
*T* stage (vs T1)	0.175			0.105
T2	0.367 (0.121–1.115)	0.077	0.341 (0.111–1.043)	0.059
T3	0.514 (0.208–1.275)	0.151	0.389 (0.151–1.004)	0.051
T4	0.547 (0.287–1.043)	0.067	0.556 (0.289–1.068)	0.078
*N* stage (vs N0)	**< 0.001**			**< 0.001**
N1	0.101 (0.028–0.369)	**< 0.001**	0.089 (0.023–0.339)	**< 0.001**
N2	0.250 (0.123–0.508)	**< 0.001**	0.217 (0.102–0.460)	**< 0.001**
N3	0.243 (0.093–0.639)	**0.004**	0.233 (0.084–0.644)	**0.005**
**IC+CCRT (vs CCRT)**	0.960 (0.667–1.382)	0.826	0.968 (0.671–1.397)	0.864
Gender (Female vs. Male)	0.945 (0.607–1.471)	0.802		
Age (>=45 vs. < 45)	2.067(1.415–3.018)	**< 0.001**	1.037(1.018–1.055)	**< 0.001**
Smoking (Yes vs. No)	1.235 (0.854–1.785)	0.262		
Drinking (Yes vs. No)	1.138(0.901–3.201)	0.608		
Family history	0.807 (0.522–1.249)	0.335		
*T* stage (vs. T1)	**< 0.001**			**0.002**
T2	0.351 (0.170–0.725)	**0.005**	0.222 (0.088–0.557)	**0.001**
T3	0.355 (0.186–0.678)	**0.002**	0.487 (0.248–0.957)	**0.037**
T4	0.413 (0.275–0.622)	**< 0.001**	0.791 (0.383–1.634)	0.526
*N* stage (vs N0)	**0.031**			**< 0.001**
N1	0.318 (0.129–0.784)	**0.013**	0.290 (0.138–0.611)	**0.001**
N2	0.561 (0.289–1.089)	0.087	0.302 (0.156–0.583)	**< 0.001**
N3	0.802 (0.391–1.647)	0.548	0.405 (0.267–0.615)	**< 0.001**

Possible prognostic factors with a *P* value < 0.1 in univariable analysis were included in the multivariable analysis together with the treatment group. OS, overall survival; HR, hazard ratio; CT, chemotherapy; AC, adjuvant chemotherapy; CCRT, concurrent chemoradiotherapy; IMRT, intensity-modulated radiotherapy; IC, induction chemotherapy. Bold values indicate *P* value < 0.05.

### 3.2 Clinical outcomes of patients treated with IMRT with or without CC

**Figure 4:**
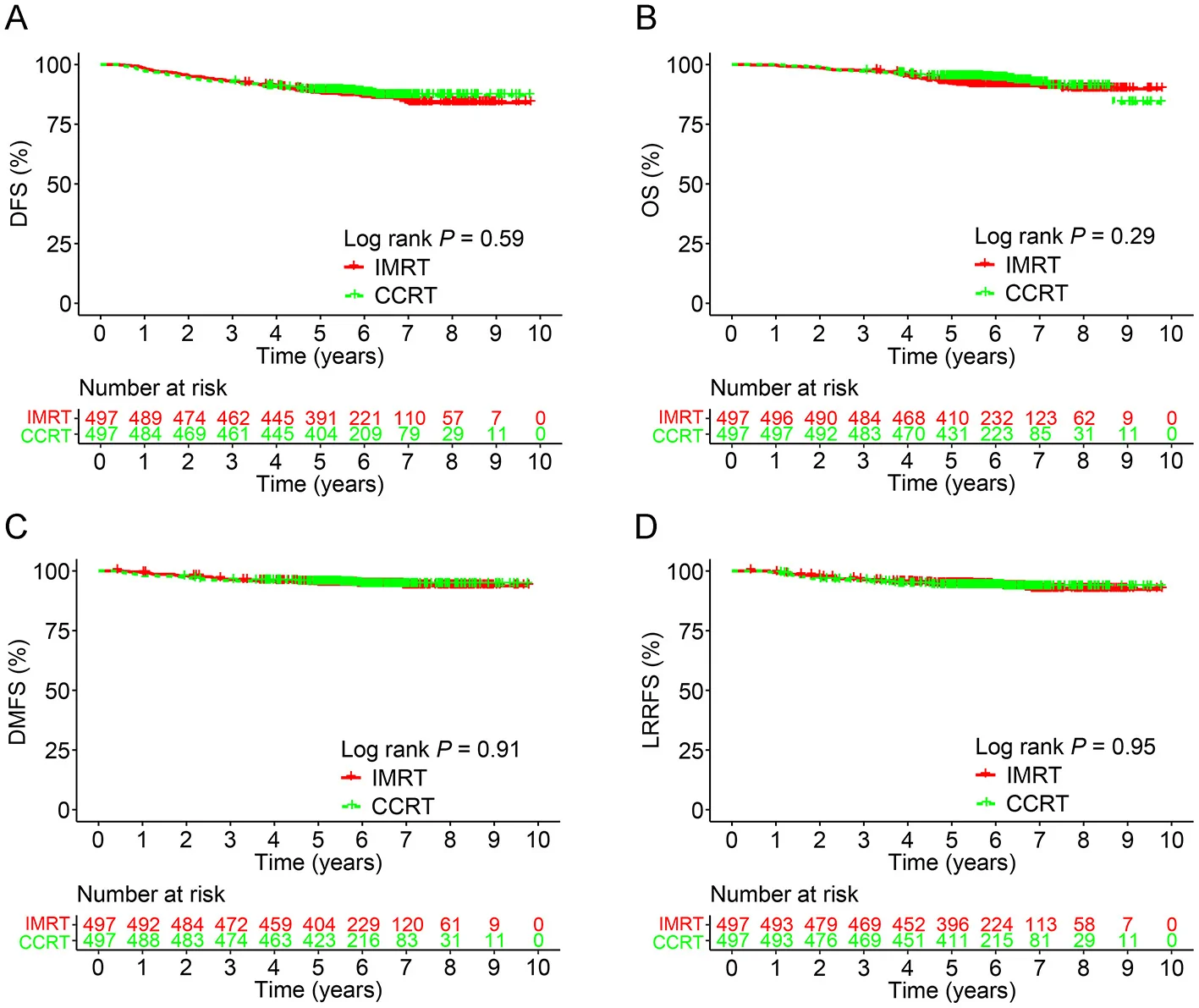
Kaplan-Meier curves for disease-free survival (DFS) **(A)**, overall survival (OS) **(B)**, distant metastasis-free survival (DMFS) **(C)**, and locoregional relapse-free survival (LRRFS) **(D)** of patients who received IMRT with or without CC.

**Figure 5:**
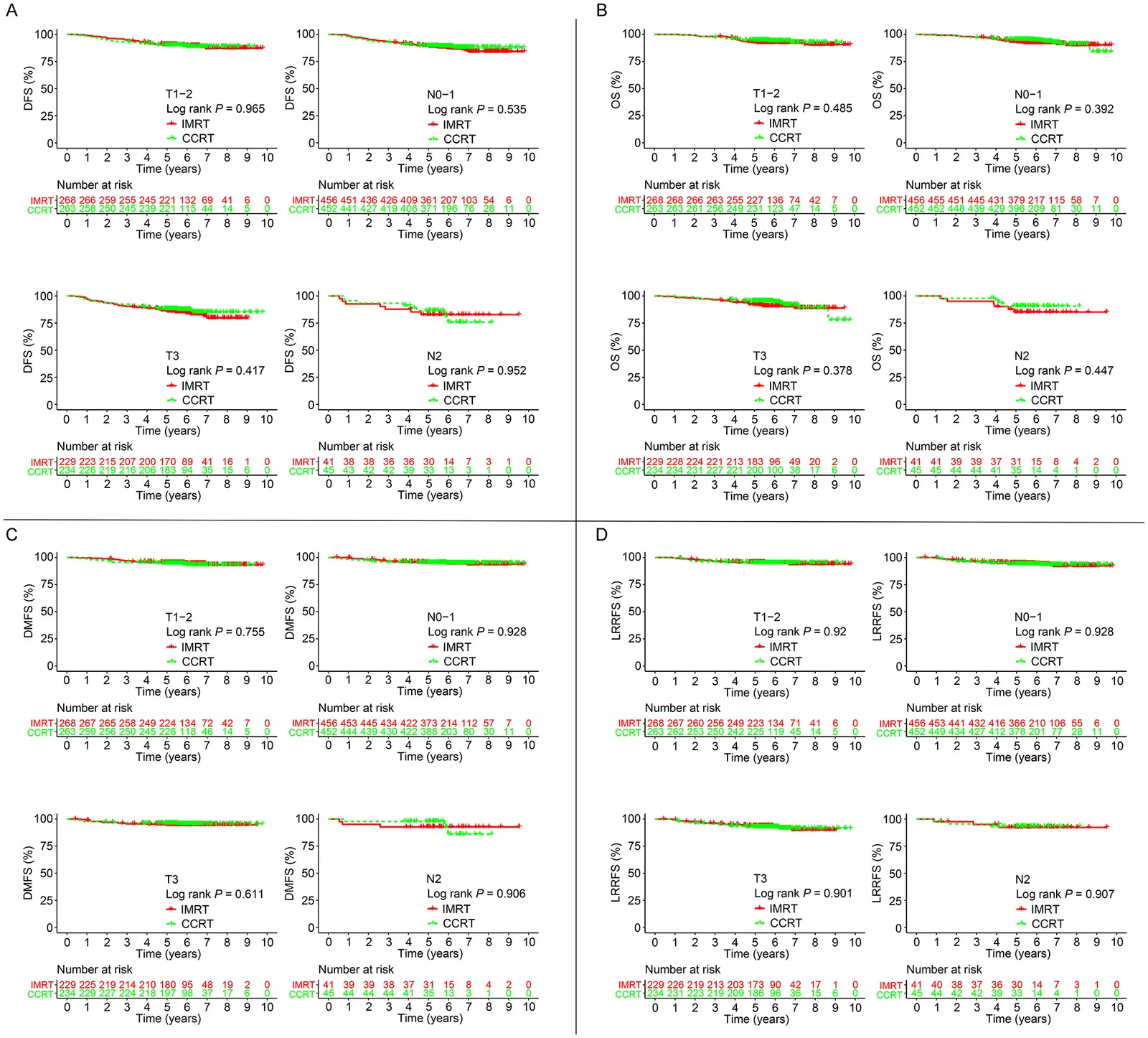
Subgroup analysis in patients with different risk stratification (i.e., T1–2, T3, N0–1, and N2) between patients receiving IMRT with or without CC for disease-free survival (DFS) **(A)**, overall survival (OS) **(B)**, distant metastasis-free survival (DMFS) **(C)**, and locoregional relapse-free survival (LRRFS) **(D)**.

A total of 497 matched pairs of patients (*n* = 994) treated with IMRT with or without CC were selected using one-to-one PSM. All of the patients exhibited well-balanced baseline characteristics, with all *P* values exceeding 0.05 (Supplementary Table S1). The 5-year DFS, OS, DMFS, and LRRFS rates for the selected 497 pairs were 88.9%, 93.7%, 95.1%, and 94.3%, respectively. The estimated 5-year DFS, OS, DMFS, and LRRFS were 89.1 and 88.6% (*P* = 0.59), 94.9 and 92.4% (*P* = 0.29), 95.1 and 94.9% (*P* = 0.91), and 93.9 and 94.7% (*P* = 0.95), respectively, in patients receiving IMRT with CC or IMRT without CC (Figures 4A-D). The multivariable analyses revealed that age and smoking were independent prognostic indicators for OS, rather than the treatment group (IMRT with CC vs. without CC) (Supplementary Table S1). No meaningful survival differences were identified between the two treatment groups when patients were categorized according to risk stratification (T1–2, T3, N0–1, and N2) (Figures 5A–D).

### 3.3 Clinical outcomes of patients treated with IC followed by IMRT with or without CC

**Figure 6:**
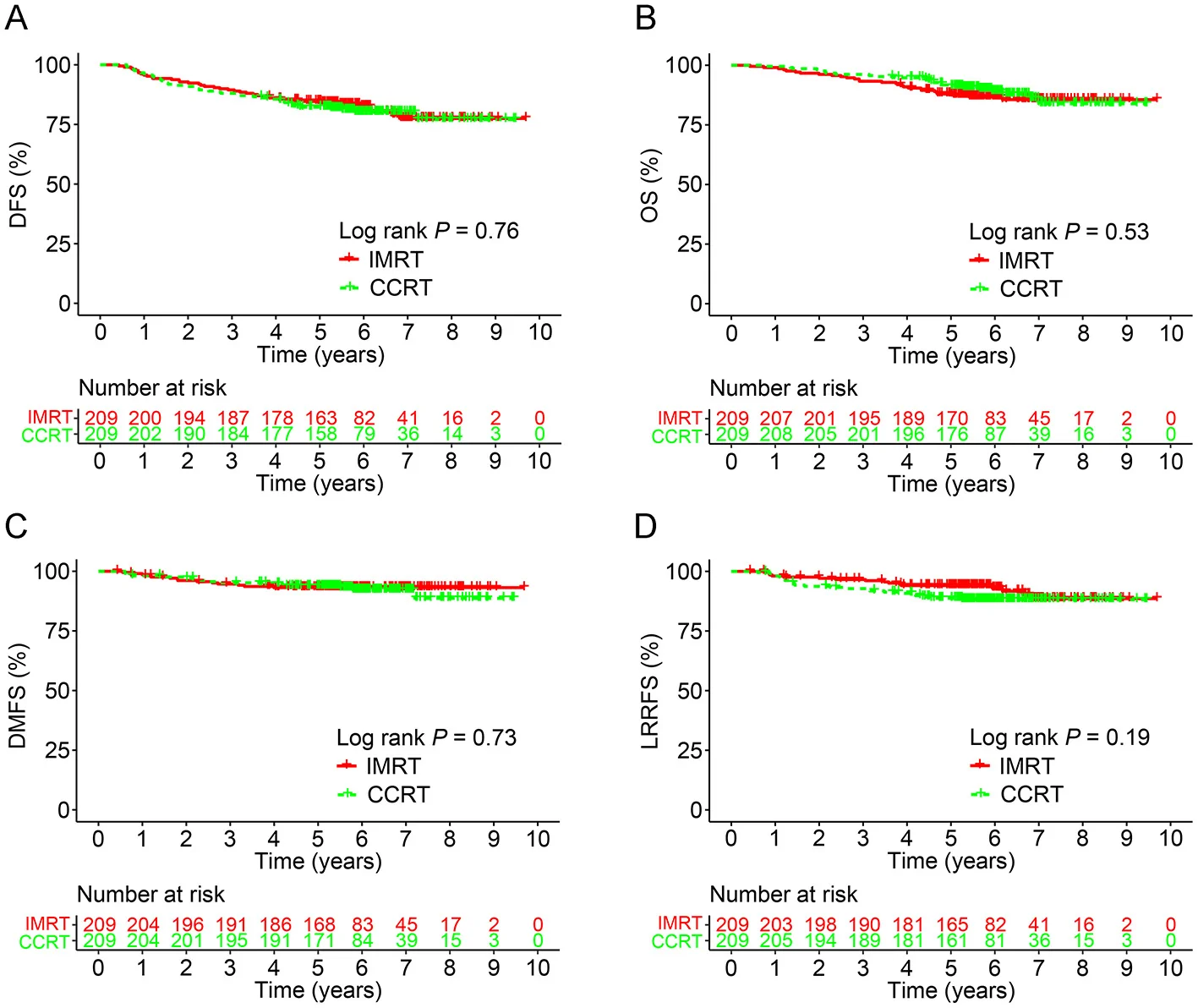
Kaplan-Meier curves for disease-free survival (DFS) **(A)**, overall survival (OS) **(B)**, distant metastasis-free survival (DMFS) **(C)**, and locoregional relapse-free survival (LRRFS) **(D)** of patients between the chemotherapy + intensity-modulated radiotherapy (CT + IMRT) and IC + CCRT groups.

**Figure 7:**
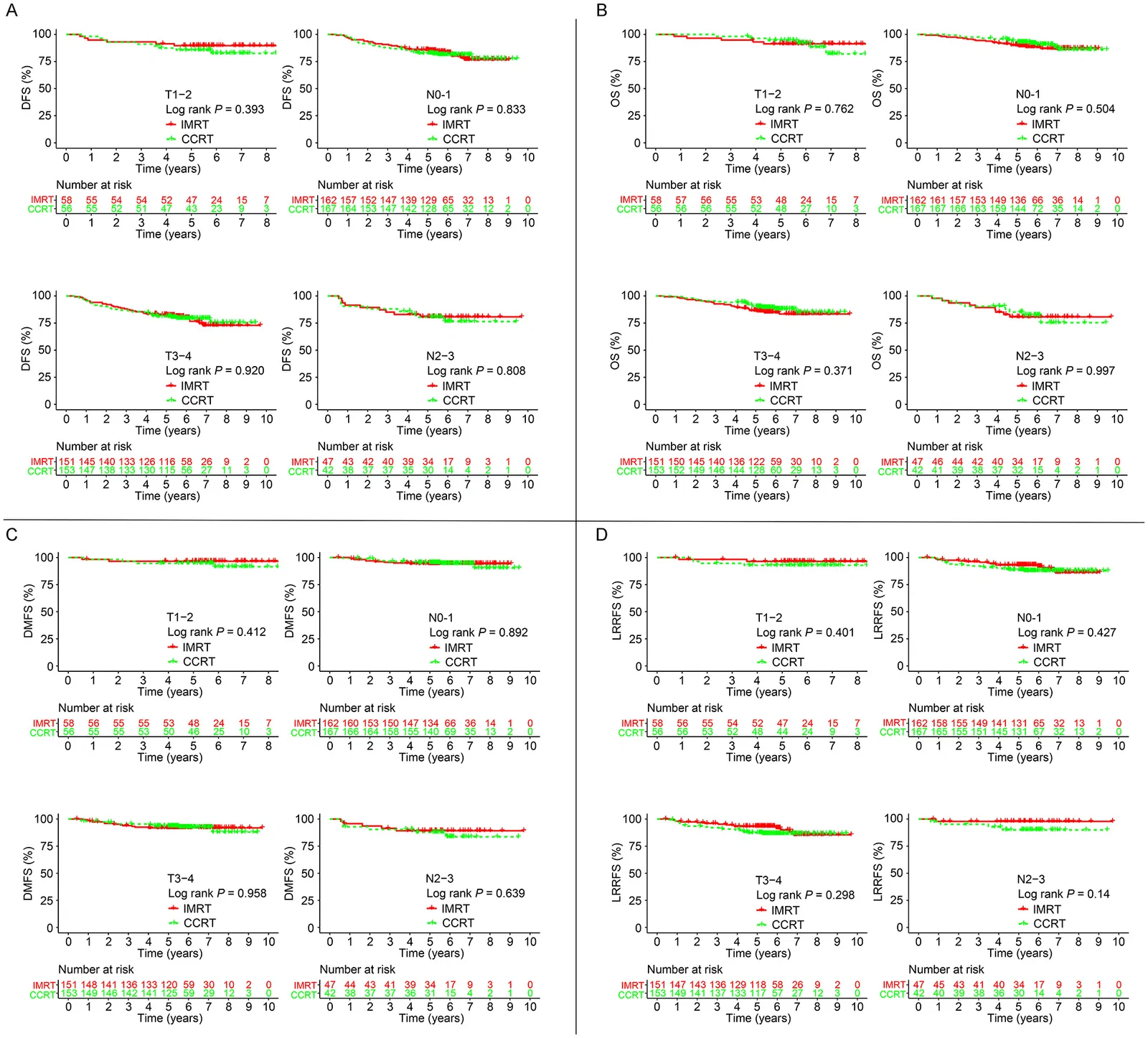
Subgroup analysis of patients based on different risk stratification (i.e., T1–2, T3–4, N0–1, and N2–3) between the chemotherapy + intensity-modulated radiotherapy (CT + IMRT) and IC + CCRT groups for disease-free survival (DFS) **(A)**, overall survival (OS) **(B)**, distant metastasis-free survival (DMFS) **(C)**, and locoregional relapse-free survival (LRRFS) **(D)**.

There were 209 pairs of patients who received IC + CCRT vs IC + IMRT (418 patients) were selected using PSM. These patients exhibited well-balanced baseline characteristics (Supplementary Table S2). The 5-year DFS, OS, DMFS, and LRRFS rates for the selected 209 pairs were 83.2%, 89.6%, 93.0%, and 91.0%, respectively. The estimated 5-year DFS, OS, DMFS, and LRRFS were 84.1 and 81.6% (*P* = 0.76), 87.4 and 91.2% (*P* = 0.53), 93.1 and 93.0% (*P* = 0.73), and 93.0 and 88.1% (*P* = 0.19), respectively, in patients receiving IC + CCRT or IC + IMRT (Figures 6A–D). Multivariable analysis revealed that *N* stage was an independent prognostic factor for OS, whereas the treatment subgroup (IC + CCRT vs. IC + IMRT) was not (Table 2). When patients were stratified by *T* and *N* categories (T1–2, T3–4, N0–1, and N2–3), no significant differences in survival outcomes were observed between the two treatment groups (Figures 7A–D).

### 3.4 Clinical outcomes of patients treated with CCRT with or without IC

**Figure 8:**
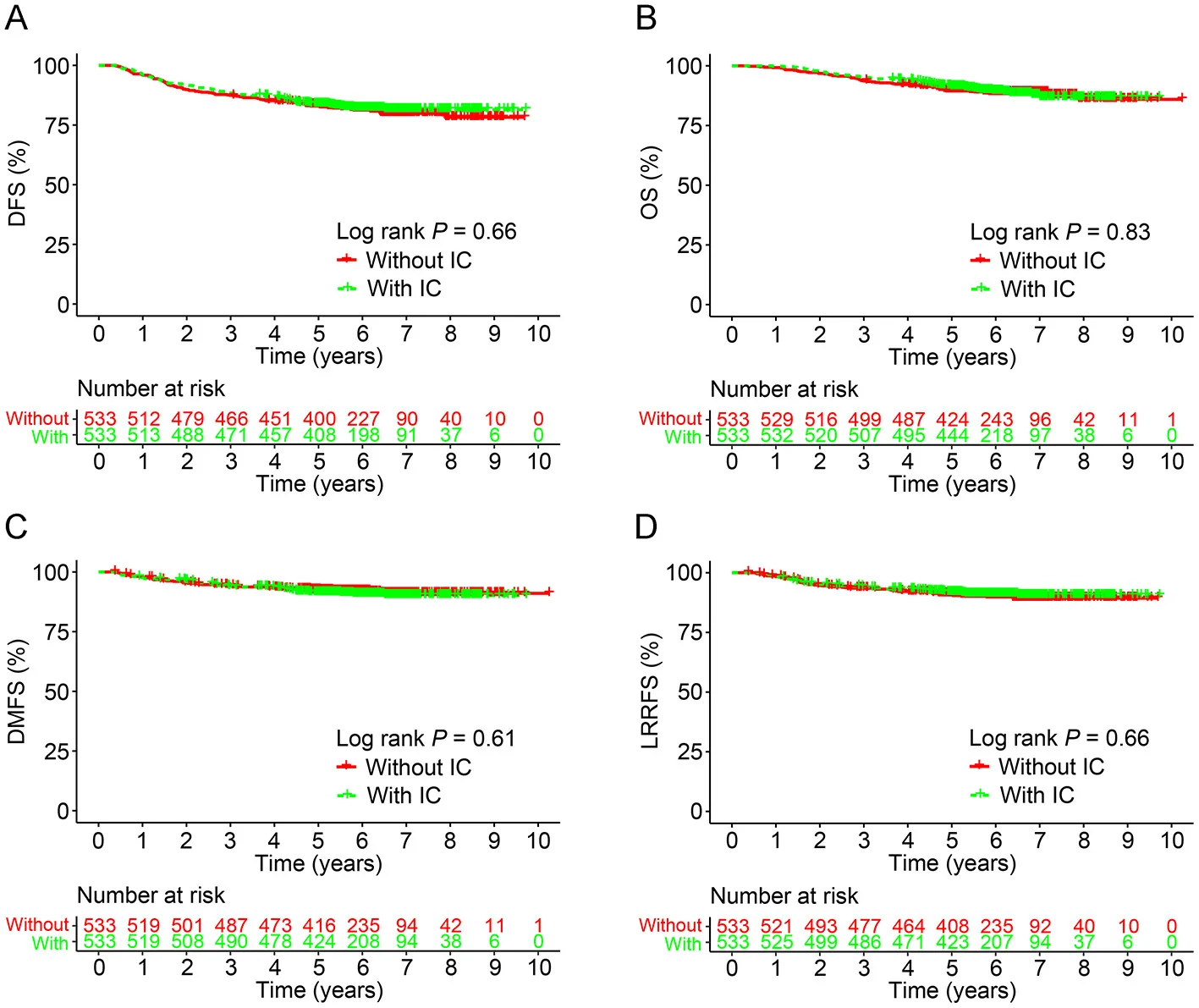
Kaplan-Meier curves for disease-free survival (DFS) **(A)**, overall survival (OS) **(B)**, distant metastasis-free survival (DMFS) **(C)**, and locoregional relapse-free survival (LRRFS) **(D)** of patients between the induction chemotherapy + concurrent chemoradiotherapy (IC + CCRT) and CCRT groups.

**Figure 9:**
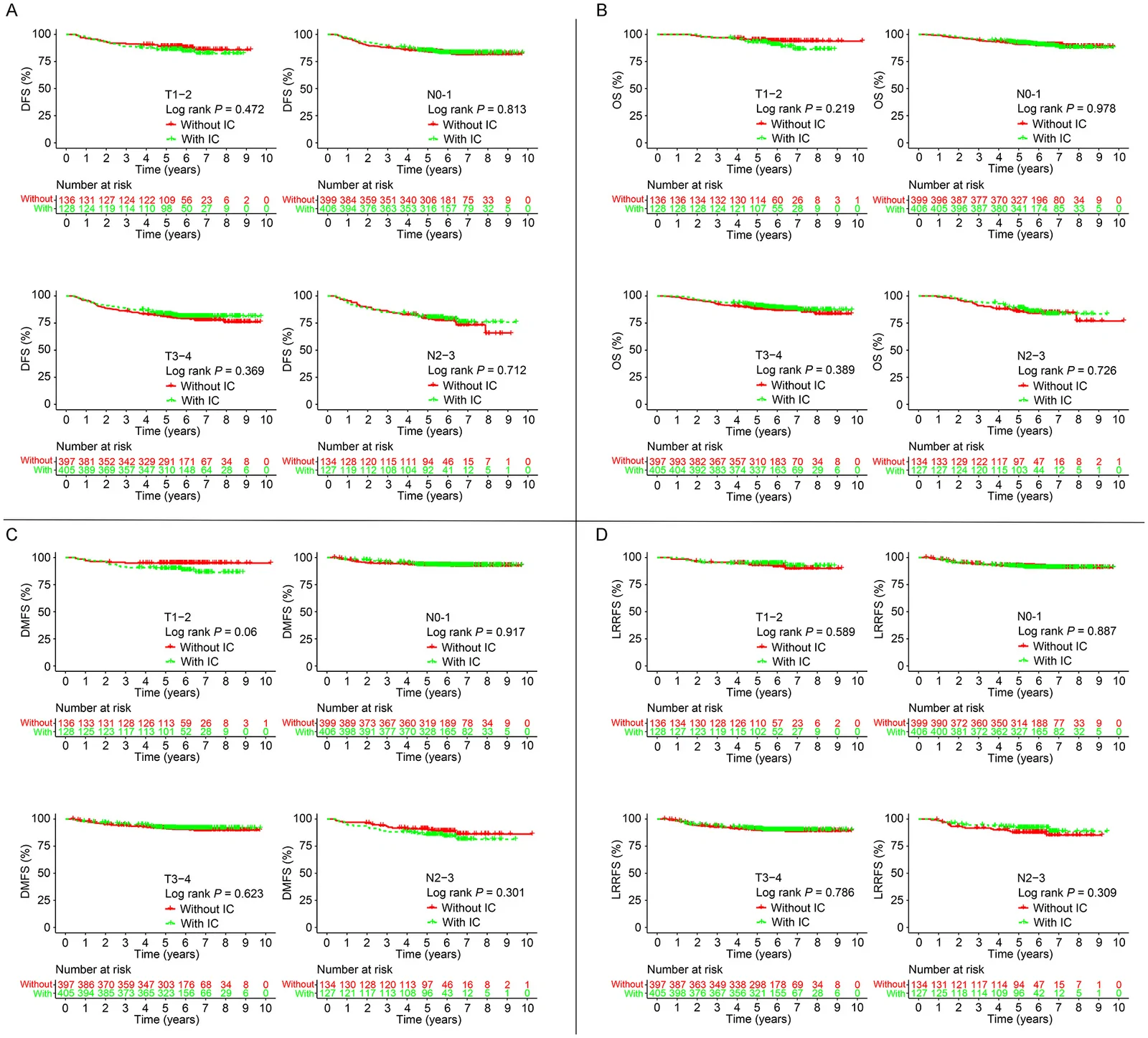
Subgroup analysis in patients with different risk stratification (i.e., T1–2, T3–4, N0–1, and N2–3) between induction chemotherapy + concurrent chemoradiotherapy (IC + CCRT) and CCRT groups for disease-free survival (DFS) **(A)**, overall survival (OS) **(B)**, distant metastasis-free survival (DMFS) **(C)**, and locoregional relapse-free survival (LRRFS) **(D)**.

A total of 533 matched pairs of patients (*n* = 1,066) who received IC + CCRT vs. CCRT were selected by PSM. After matching, the baseline characteristics were well balanced between the two groups (Supplementary Table S3). The 5-year DFS, OS, DMFS, and LRRFS rates for the selected 533 pairs were 91.8%, 90.6%, 83.5%, and 91.3%, respectively. The estimated 5-year DFS, OS, DMFS, and LRRFS were 82.6 and 83.2% (*P* = 0.66), 91.3 and 90.0% (*P* = 0.83), 91.4 and 92.3% (*P* = 0.61), and 91.7 and 90.9% (*P* = 0.66), respectively, in patients receiving IC + CCRT or CCRT alone (Figures 8A–D). In multivariable analysis, age, *T* stage, and *N* stage were independent prognostic indicators of OS, while no significant prognostic effect was observed for the treatment subgroup (IC + CCRT vs. CCRT) (Table 2). Consistently, subgroup analyses across different risk strata (T1–2, T3–4, N0–1, and N2–3) did not reveal significant survival differences between the two treatment groups (Figures 9A–D).

### 3.5 Clinical outcomes of patients treated with CCRT with or without AC

**Figure 10:**
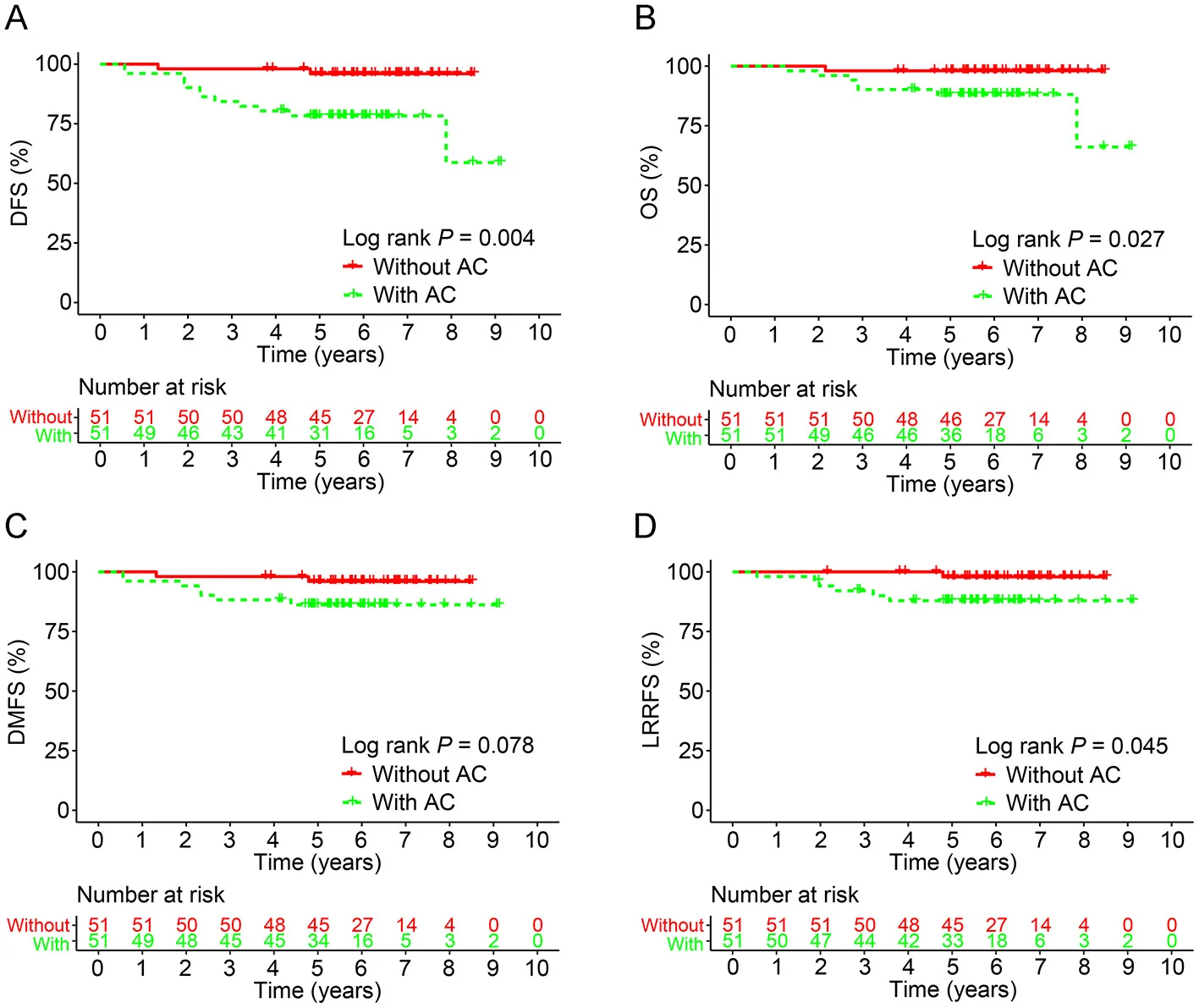
Kaplan-Meier curves for disease-free survival (DFS) **(A)**, overall survival (OS) **(B)**, distant metastasis-free survival (DMFS) **(C)**, and locoregional relapse-free survival (LRRFS) **(D)** of patients between the CCRT + AC and CCRT groups.

A total of 51 matched pairs of patients (*n* = 102) receiving CCRT + AC vs. CCRT were selected by PSM, and IC was permitted in both groups. Baseline characteristics were well balanced between the two groups (Supplementary Table S4). The 5-year DFS, OS, DMFS, and LRRFS rates for the selected 51 pairs were 87.1%, 93.1%, 91.0%, and 92.9%, respectively. Survival analysis showed that CCRT was associated with significantly improved DFS, OS, and LRRFS compared with CCRT + AC. The estimated 5-year DFS, OS, and LRRFS rates were 96.0% vs. 78.3% (*P* = 0.004), 98.0% vs. 88.1% (*P* = 0.027), and 97.9% vs. 87.9% (*P* = 0.045), respectively. Although the difference in DMFS did not reach statistical significance, a borderline significance was observed in favor of the CCRT group (96.0% vs. 86.1%, *P* = 0.078). (Figures 10A–D).

The data from the 102 patients could not fit in the multivariate analysis due to the small sample size.

## 4 Discussion

Since the discovery of the effect of CT in addition to RT on II–IVA NPC based on the results of clinical trials (22, 23), a large number of chemoradiotherapy regimens have been reported to improve patient survival rates (e.g., IC, CC, and AC). However, these clinical trials included a higher percentage of patients with detectable pre-DNA. Whether these results apply to patients with undetectable pre-DNA remains unclear. This is the first study to explore the association between multiple treatment modalities and the long-term clinical outcomes of II–IVA NPC patients with undetectable pre-DNA. In our cohort, among NPC patients with undetectable pre-DNA, the addition of chemotherapy to IMRT was not associated with statistically significant improvements in DFS, OS, DMFS, or LRRFS when compared with IMRT alone, regardless of whether IC, CC, or AC was administered.

The 5-year DFS, OS, DMFS, and LRRFS observed in our study were higher than those reported in historical controls, which may be partly attributable to the inclusion of a large proportion of NPC patients with undetectable pre-DNA. In the study by Du et al., (24) the reported 5-year FFS, OS, DMFS, and LRRFS rates for patients with stage I–IVA NPC ranged from 77.7 to 79.4%, 84.8 to 88.6%, 86.6 to 87.3%, and 88.5 to 89.9%, respectively, with approximately 96% of patients having stage II–IVA disease. Notably, the study population consisted exclusively of EBV-related NPC patients. Previous studies have consistently reported an association between low EBV-DNA levels and a favorable prognosis in patients with NPC (3, 25, 26). Li et al. (25) categorized patients according to EBV DNA status before and 3 months post-treatment and observed that those with undetectable EBV DNA at both time points demonstrated markedly better 5-year PFS (81.4%), DMFS (87.3%), LRFS (92.3%), and OS (93.5%) than patients with detectable EBV DNA either before or after treatment. In the study by Nicholls et al., (27) plasma EBV DNA-negative patients (plasma EBV DNA ≤ 20 copies/mL) demonstrated significantly improved 5-year PFS compared with EBV DNA-positive patients (92.7% vs. 70.0%, *P* = 0.023), while a trend toward better 5-year OS was also observed, although the difference was not statistically significant (91.9% vs. 78.1%, *P* = 0.242). It should be noted that patients with stage II–IVA disease accounted for 76.9% of the EBV DNA-negative cohort and that the sample size of plasma EBV DNA-negative patients was relatively small, comprising only 78 patients. Another study including 325 patients with EBV DNA-negative NPC (cutoff level: 500 copies/mL) across stages I–IVB reported 5-year PFS and OS rates ranging from 78.8 to 80.6% and from 87.7 to 89.4%, respectively (28). Similarly, Weng et al. (26) analyzed 257 patients with stage I–IVA NPC who had undetectable EBV DNA (0 copies/mL) during treatment and reported favorable long-term outcomes, with 5-year OS, LRRFS, DMFS, and PFS rates of 88.72, 90.66, 91.44, and 84.05%, respectively. Taken together, these findings consistently suggest that NPC patients with undetectable or low EBV DNA levels tend to have more favorable survival outcomes.

Some studies have yielded the opposite results. The addition of IC to CCRT revealed a better 5-year DFS (77.4% vs. 66.4%; *P* = 0.019), OS (OS, 85.6% vs. 77.7%; *P* = 0.042), DMFS (88% vs. 79.8%; *P* = 0.030), and LRRFS (90.7% vs. 83.8%; *P* = 0.044) compared to the group that received CCRT alone (5). Th addition of CC to IMRT improved the 3-year OS of patients with pre-DNA levels > 1,460 copies/mL. The addition of CC to IMRT improved the 3-year DFS (85.5% vs. 78.3%; *P* = 0.043) and OS (94.3% vs.88.5%; *P* = 0.003) of patients with pre-DNA levels > 1,460 copies/mL (13). However, a study by Dai et al. (29) demonstrated that in patients with locoregionally advanced NPC, radiotherapy alone following IC with the TPF regimen achieved a comparable 3-year PFS, while being associated with a lower incidence of grade 3–4 acute toxicities. Our findings were partially inconsistent with those of previous studies, which may be attributable to the fact that our cohort exclusively included patients with undetectable pre-DNA. However, the pre-DNA concentrations in the other study were higher (IQR: 626 – 33,400)(5).

Regarding the role of AC, Chen et al. (30) evaluated the efficacy of adjuvant PF chemotherapy (cisplatin plus fluorouracil) following CCRT in patients with stage III–IV NPC (excluding T3–4N0 disease) and reported no significant improvements in failure-free survival (FFS), OS, DMFS, or LRFS. In contrast, a large randomized controlled trial investigating adjuvant metronomic capecitabine in patients with stage III–IVA disease (excluding T3–4N0 and T3N1 disease) demonstrated significant improvements in 3-year FFS, OS, DMFS, and LRFS (31). Notably, 42% (87/205) of patients experienced grade 3–4 toxicities during the PF-based AC period, whereas only 17% (35/201) of patients treated with metronomic capecitabine developed grade 3–4 adverse events across the entire treatment period (30, 31), indicating superior tolerability and lower toxicity with metronomic chemotherapy. Consistently, several retrospective studies have also reported survival benefits with metronomic AC in patients with high-risk locoregionally advanced NPC (32–34). Twu et al. (35) demonstrated that adjuvant oral tegafur–uracil reduced distant failure and improved OS in patients with persistently detectable plasma EBV DNA following definitive chemoradiotherapy.

However, neither of the aforementioned studies performed stratified analyses according to pre-treatment EBV DNA status. To date, evidence regarding the role of AC in NPC patients with undetectable pre-DNA remains scarce. In the present study, no survival advantage was observed with the addition of AC to CCRT. These findings suggest that the effectiveness of AC may be context-dependent and influenced by multiple clinical and biological factors, such as tumor stage, EBV DNA status, and chemotherapy regimens, including metronomic chemotherapy. Therefore, future studies are warranted to further identify the NPC subgroups most likely to benefit from AC and to explore more optimal AC strategies.

Taken together, several factors may account for our negative findings. First, patients with undetectable pre-DNA levels are at a relatively low risk, and the incremental benefit of CT may be limited. Prior research from our institution has suggested that, among patients with low-risk NPC, the addition of CC did not confer a significant survival benefit, whereas IMRT alone was associated with a significantly lower incidence of grade 3–4 adverse events, including hematologic and nonhematologic toxicities (36, 37). Similarly, compared with IC + IMRT alone, IC + CCRT was associated with a significantly higher incidence of grade 3–4 toxicities (29), and the addition of IC before CCRT also resulted in significantly more severe toxicities than CCRT alone (38). Moreover, the incorporation of IC was reported to compromise the tolerability of subsequent CC (38). And it has previously been reported that severe treatment-associated lymphocytopenia is related to poor PFS for squamous cell head and neck cancer patients (39). In addition, it is important to note that more than 5% weight loss was observed in 30% of the patients in the CCRT group, which was identified to be an independent factor related to low survival rate, resulting in adverse treatment effects, including compliance and poor postural accuracy for IMRT (40–42). Although these toxicities appeared to be well-tolerated, they might have further reduced the therapeutic effect of CT in this population. In addition, several factors (e.g., genetic, molecular, biological, and other non-anatomical factors) can likely influence patient prognosis (40). In the future, we need to explore the molecular mechanism of with undetectable pre-DNA and insensitivity to chemotherapy.

In the multivariable analysis, age, smoking status, and N stage were identified as independent prognostic factors for survival, which is consistent with previous reports (30, 36, 43). In particular, advanced N stage has been well recognized as an indicator of a higher risk of distant metastasis and poorer prognosis, and in clinical practice is often considered when selecting more intensive chemotherapy strategies. However, in the present study, subgroup analyses according to N stage showed that the addition of chemotherapy did not confer a survival benefit in patients with more advanced nodal disease. This finding may be partly explained by the distinct biological characteristics and relatively lower chemosensitivity of patients with undetectable pre-DNA, which may differ from those with EBV-related NPC. Furthermore, in our cohort, radiotherapy alone was associated with significantly improved LRRFS compared with IMRT plus chemotherapy in the T1–2 subgroup. This observation may be attributable to potential delays in the initiation of definitive IMRT caused by IC, as well as chemotherapy-related toxicities that could compromise the therapeutic efficacy of radiotherapy.

The present findings provide previously unknown evidence regarding comprehensive treatment patterns in stage II–IVA NPC patients with undetectable pre-treatment EBV DNA. We hope these findings will eventually inform individualized treatment decisions after confirmation in prospective randomized trials. For this specific population, our results suggest that CT may be safely omitted, potentially minimizing treatment-related toxicities without compromising survival outcomes, especially among patients with T1–2 disease. However, these findings require prospective validation.

There were several limitations associated with this study. First, this is a retrospective study with unavoidable selection bias; therefore, we performed PSM to minimize this bias. Second, since all patients were from a single treatment center, these preliminary findings require validation in prospective, multi-institutional studies, including adequately powered randomized controlled trials. Third, the lack of detailed toxicity data limited our ability to comprehensively assess treatment-related adverse events and safety outcomes. Furthermore, selection bias may exist specifically for patients who received radiotherapy alone, as those receiving IMRT alone were not randomly assigned but selected based on individual clinical judgment. Although we applied strict inclusion criteria and propensity score matching to balance measured covariates, unmeasured confounding remains. The potential direction of bias is uncertain: if clinicians preferentially omitted chemotherapy in patients with low-risk disease features (e.g., early stage or favorable biomarkers), the IMRT-alone group would have a prognostic advantage, potentially biasing the comparison toward a false non-inferiority conclusion; conversely, if omission occurred in patients who were frailer or had comorbidities, bias could favor the chemoradiotherapy group. Therefore, our finding of no statistically significant difference in prognosis between the two groups should be interpreted with caution.

In conclusion, our analysis did not detect a statistically significant survival benefit associated with the addition of chemotherapy (induction, concurrent, or adjuvant) to IMRT in stage II–IVA NPC patients with undetectable pre-treatment EBV DNA. These preliminary findings suggest that IMRT alone may warrant further investigation as a potential treatment option. However, given the observational study design, validation in prospective randomized trials is required before these results can inform individualized treatment decision-making.

## Data Availability

The data analyzed in this study is subject to the following licenses/restrictions: The original contributions presented in the study are included in the article/Supplementary material, further inquiries can be directed to the corresponding authors. Requests to access these datasets should be directed to Qing-qing Xu xuqq@sysucc.org.cn.

## References

[1] Chua MLK Wee JTS Hui EP Chan ATC. Nasopharyngeal carcinoma. Lancet. (2016) 387:1012–24. doi: 10.1016/S0140-6736(15)00055-0 26321262

[2] Yu MC Yuan J-M. Epidemiology of nasopharyngeal carcinoma. Semin Cancer Biol. (2002) 12:421–9. doi: 10.1016/S1044579X02000858 12450728

[3] Lin J-C Wang W-Y Chen KY Wei Y-H Liang W-M Jan J-S . Quantification of plasma Epstein-Barr virus DNA in patients with advanced nasopharyngeal carcinoma. N Engl J Med. (2004) 350:2461–70. doi: 10.1056/NEJMoa032260 15190138

[4] Shotelersuk K Khorprasert C Sakdikul S Pornthanakasem W Voravud N Mutirangura A. Epstein-Barr virus DNA in serum/plasma as a tumor marker for nasopharyngeal cancer. Clin Cancer Res. (2000) 6:1046–51. 10741733

[5] Li WF Chen NY Zhang N Hu GQ Xie FY Sun Y . Concurrent chemoradiotherapy with/without induction chemotherapy in locoregionally advanced nasopharyngeal carcinoma: long-term results of phase 3 randomized controlled trial. Int J Cancer. (2019) 145:295–305. doi: 10.1002/ijc.32099 30613964

[6] Guo R Tang L-L Mao Y-P Du X-J Chen L Zhang Z-C . Proposed modifications and incorporation of plasma Epstein-Barr virus DNA improve the TNM staging system for Epstein-Barr virus-related nasopharyngeal carcinoma. Cancer. (2019) 125:79–89. doi: 10.1002/cncr.31741 30351466

[7] Au KH Ngan RKC Ng AWY Poon DMC Ng WT Yuen KT . Treatment outcomes of nasopharyngeal carcinoma in modern era after intensity modulated radiotherapy (IMRT) in Hong Kong: a report of 3328 patients (HKNPCSG 1301 study). Oral Oncol. (2018) 77:16–21. doi: 10.1016/j.oraloncology.2017.12.004 29362121

[8] Mao YP Tang LL Chen L Sun Y Qi ZY Zhou GQ . Prognostic factors and failure patterns in non-metastatic nasopharyngeal carcinoma after intensity-modulated radiotherapy. Chin J Cancer. (2016) 35:103. doi: 10.1186/s40880-016-0167-2 28031050 PMC5192583

[9] Ng WT Tung SY Lee V Ngan RKC Choi HCW Chan LLK . Concurrent-adjuvant chemoradiation therapy for stage III-IVB nasopharyngeal carcinoma-exploration for achieving optimal 10-Year therapeutic ratio. Int J Radiat Oncol Biol Phys. (2018) 101:1078–86. doi: 10.1016/j.ijrobp.2018.04.069 29885997

[10] Sun Y Li WF Chen NY Zhang N Hu GQ Xie FY . Induction chemotherapy plus concurrent chemoradiotherapy versus concurrent chemoradiotherapy alone in locoregionally advanced nasopharyngeal carcinoma: a phase 3, multicentre, randomised controlled trial. Lancet Oncol. (2016) 17:1509–20. doi: 10.1016/S1470-2045(16)30410-7 27686945

[11] Yang Q Zhao TT Qiang MY Hu L Lv X Ye YF . Concurrent chemoradiotherapy versus intensity-modulated radiotherapy alone for elderly nasopharyngeal carcinoma patients with pre-treatment Epstein-Barr virus DNA: a cohort study in an endemic area with long-term follow-up. J Cancer. (2018) 9:3023–31. doi: 10.7150/jca.26145 30210624 PMC6134827

[12] Colevas AD Yom SS Pfister DG Spencer S Adelstein D Adkins D . NCCN guidelines insights: head and neck cancers, version 1.2018. J Natl Compr Canc Netw. (2018) 16:479–90. doi: 10.6004/jnccn.2018.0026 29752322

[13] Sun XS Chen WH Liu SL Liang YJ Chen QY Guo SS . Individualized concurrent chemotherapy by pretreatment plasma Epstein-Barr viral DNA in II-III stage nasopharyngeal carcinoma: a propensity score matching analysis using a large cohort. Cancer Med. (2019) 8:4214–25. doi: 10.1002/cam4.2343 31210417 PMC6675745

[14] Wei ZG Hu XL He Y Guan H Wang JJ He L . Clinical and survival analysis of nasopharyngeal carcinoma with consistently negative Epstein-Barr virus DNA. Head Neck. (2021) 43:1465–75. doi: 10.1002/hed.26608 33421240

[15] Alfieri S Romano R Marceglia S Sciortino C Ferrari Bravo W Zucchini M . Longitudinal assessment of plasma EBV-DNA in non-endemic EBV-related nasopharyngeal cancer (NPC): the LEA study. Eur J Cancer. (2025) 226:115625. doi: 10.1016/j.ejca.2025.115625 40729842

[16] Alfieri S Iacovelli NA Marceglia S Lasorsa I Resteghini C Taverna F . Circulating pre-treatment Epstein-Barr virus DNA as prognostic factor in locally-advanced nasopharyngeal cancer in a non-endemic area[J]. Oncotarget. (2017) 8:47780–9. doi: 10.18632/oncotarget.17822 28562354 PMC5564604

[17] Huynh C Walsh LA Spicer JD. Surgery after neoadjuvant immunotherapy in patients with resectable non-small cell lung cancer. Transl Lung Cancer Res. (2021) 10:563–80. doi: 10.21037/tlcr-20-509 33569337 PMC7867741

[18] Shao J-Y Li Y-H Gao H-Y Wu Q-L Cui N-J Zhang L . Comparison of plasma Epstein-Barr virus (EBV) DNA levels and serum EBV immunoglobulin A/virus capsid antigen antibody titers in patients with nasopharyngeal carcinoma. Cancer. (2004) 100:1162–70. doi: 10.1002/cncr.20099 15022282

[19] Lee AWM Lin JC Ng WT. Current management of nasopharyngeal cancer. Semin Radiat Oncol. (2012) 22:233–44. doi: 10.1016/j.semradonc.2012.03.008 22687948

[20] Tang L-Q Chen D-P Guo L Mo H-Y Huang Y Guo S-S . Concurrent chemoradiotherapy with nedaplatin versus cisplatin in stage II-IVB nasopharyngeal carcinoma: an open-label, non-inferiority, randomised phase 3 trial. Lancet Oncol. (2018) 19:461–73. doi: 10.1016/S1470-2045(18)30104-9 29501366

[21] Afqir S Ismaili N Errihani H. Concurrent chemoradiotherapy in the management of advanced nasopharyngeal carcinoma: current status. J Cancer Res Ther. (2009) 5:3–7. doi: 10.4103/0973-1482.48763 19293481

[22] Yang Q Cao S-M Guo L Hua Y-J Huang P-Y Zhang X-L . Induction chemotherapy followed by concurrent chemoradiotherapy versus concurrent chemoradiotherapy alone in locoregionally advanced nasopharyngeal carcinoma: long-term results of a phase III multicentre randomised controlled trial. Eur J Cancer. (2019) 119:87–96. doi: 10.1016/j.ejca.2019.07.007 31425966

[23] Li W-F Chen L Sun Y Ma J. Induction chemotherapy for locoregionally advanced nasopharyngeal carcinoma. Chin J Cancer. (2016) 35:94. doi: 10.1186/s40880-016-0157-4 27846913 PMC5111216

[24] Du XJ Wang GY Zhu XD Han YQ Lei F Shen LF . Refining the 8th edition TNM classification for EBV related nasopharyngeal carcinoma. Cancer Cell. (2024) 42:464–73 e3. doi: 10.1016/j.ccell.2023.12.020 38242125

[25] Li W Yang C Lv Z Li J Li Z Yuan X . Integrating pre- and post-treatment Plasma Epstein-Barr Virus DNA levels for better prognostic prediction of Nasopharyngeal Carcinoma. J Cancer. (2021) 12:2715–22. doi: 10.7150/jca.56397 33854631 PMC8040726

[26] Weng Y Wu L Li Y Wang J Wu Z Hong X . Prognostic value of immune-inflammatory and nutrition indicators in non-metastatic nasopharyngeal carcinoma with negative plasma Epstein-Barr virus DNA. Ther Adv Med Oncol. (2024) 16:17588359241286489. doi: 10.1177/17588359241286489 39403452 PMC11472370

[27] Nicholls JM Lee VH Chan SK Tsang KC Choi CW Kwong DL . Negative plasma Epstein-Barr virus DNA nasopharyngeal carcinoma in an endemic region and its influence on liquid biopsy screening programmes. Br J Cancer. (2019) 121:690–8. doi: 10.1038/s41416-019-0575-6 31527689 PMC6888810

[28] Yuan X Yang H Zeng F Zhou S Wu S Yuan Y . Prognostic value of systemic inflammation response index in nasopharyngeal carcinoma with negative Epstein-Barr virus DNA. BMC Cancer. (2022) 22:858. doi: 10.1186/s12885-022-09942-1 35932022 PMC9356473

[29] Dai J Zhang B Su Y Pan Y Ye Z Cai R . Induction chemotherapy followed by radiotherapy vs. chemoradiotherapy in nasopharyngeal carcinoma: a randomized clinical trial. JAMA Oncol. (2024) 10:456–63. doi: 10.1001/jamaoncol.2023.6552 38329737 PMC10853870

[30] Chen L Hu CS Chen XZ Hu GQ Cheng ZB Sun Y . Concurrent chemoradiotherapy plus adjuvant chemotherapy versus concurrent chemoradiotherapy alone in patients with locoregionally advanced nasopharyngeal carcinoma: a phase 3 multicentre randomised controlled trial. Lancet Oncol. (2012) 13:163–71. doi: 10.1016/S1470-2045(11)70320-5 22154591

[31] Chen YP Liu X Zhou Q Yang KY Jin F Zhu XD . Metronomic capecitabine as adjuvant therapy in locoregionally advanced nasopharyngeal carcinoma: a multicentre, open-label, parallel-group, randomised, controlled, phase 3 trial. Lancet. (2021) 398:303–13. doi: 10.1016/S0140-6736(21)01123-5 34111416

[32] Chen JH Huang WY Ho CL Chao TY Lee JC. Evaluation of oral tegafur-uracil as metronomic therapy following concurrent chemoradiotherapy in patients with non-distant metastatic TNM stage IV nasopharyngeal carcinoma. Head Neck. (2019) 41:3775–82. doi: 10.1002/hed.25904 31435974

[33] Liu YC Wang WY Twu CW Jiang RS Liang KL Wu CT . Prognostic impact of adjuvant chemotherapy in high-risk nasopharyngeal carcinoma patients. Oral Oncol. (2017) 64:15–21. doi: 10.1016/j.oraloncology.2016.11.008 28024719

[34] Yu YF Wu P Zhuo R Wu SG. Metronomic S-1 adjuvant chemotherapy improves survival in patients with locoregionally advanced nasopharyngeal carcinoma. Cancer Res Treat. (2024) 56:1058–67. doi: 10.4143/crt.2023.1343 38374697 PMC11491245

[35] Twu CW Wang WY Chen CC Liang KL Jiang RS Wu CT . Metronomic adjuvant chemotherapy improves treatment outcome in nasopharyngeal carcinoma patients with postradiation persistently detectable plasma Epstein-Barr virus deoxyribonucleic acid. Int J Radiat Oncol Biol Phys. (2014) 89:21–9. doi: 10.1016/j.ijrobp.2014.01.052 24725686

[36] Zhang WW Lin JY Wang GY Huang CL Tang LL Mao YP . Radiotherapy alone versus concurrent chemoradiotherapy in patients with stage II and T3N0 nasopharyngeal carcinoma with adverse features: a propensity score-matched cohort study. Radiother Oncol. (2024) 194:110189. doi: 10.1016/j.radonc.2024.110189 38432309

[37] Tang LL Guo R Zhang N Deng B Chen L Cheng ZB . Effect of radiotherapy alone vs radiotherapy with concurrent chemoradiotherapy on survival without disease relapse in patients with low-risk nasopharyngeal carcinoma: a randomized clinical trial. JAMA. (2022) 328:728–36. doi: 10.1001/jama.2022.13997 35997729 PMC9399866

[38] Cao SM Yang Q Guo L Mai HQ Mo HY Cao KJ . Neoadjuvant chemotherapy followed by concurrent chemoradiotherapy versus concurrent chemoradiotherapy alone in locoregionally advanced nasopharyngeal carcinoma: a phase III multicentre randomised controlled trial. Eur J Cancer. (2017) 75:14–23. doi: 10.1016/j.ejca.2016.12.039 28214653

[39] Campian JL Sarai G Ye X Marur S Grossman SA. Association between severe treatment-related lymphopenia and progression-free survival in patients with newly diagnosed squamous cell head and neck cancer. Head Neck. (2014) 36:1747–53. doi: 10.1002/hed.23535 24174270 PMC4081494

[40] Liu N Chen NY Cui RX Li WF Li Y Wei RR . Prognostic value of a microRNA signature in nasopharyngeal carcinoma: a microRNA expression analysis. Lancet Oncol. (2012) 13:633–41. doi: 10.1016/S1470-2045(12)70102-X 22560814

[41] Leung SF Chan AT Zee B Ma B Chan LY Johnson PJ . Pretherapy quantitative measurement of circulating Epstein-Barr virus DNA is predictive of posttherapy distant failure in patients with early-stage nasopharyngeal carcinoma of undifferentiated type. Cancer. (2003) 98:288–91. doi: 10.1002/cncr.11496 12872347

[42] Shen LJ Chen C Li BF Gao J Xia YF. High weight loss during radiation treatment changes the prognosis in under-/normal weight nasopharyngeal carcinoma patients for the worse: a retrospective analysis of 2433 cases. PLoS One. (2013) 8:e68660. doi: 10.1371/journal.pone.0068660 23869226 PMC3711826

[43] Chen L Zhang Y Lai SZ Li WF Hu WH Sun R . 10-Year results of therapeutic ratio by intensity-modulated radiotherapy versus two-dimensional radiotherapy in patients with nasopharyngeal carcinoma. Oncologist. (2019) 24:e38–45. doi: 10.1634/theoncologist.2017-0577 30082487 PMC6324627

