# Supplementary material for: Treatment and clinical outcomes among stage II–IVA nasopharyngeal carcinoma patients with undetectable pretreatment Epstein-Barr viral DNA
Source: Front Med (Lausanne). 2026 Aug 27;13:1752053. doi: 10.3389/fmed.2026.1752053 (PMC13558178; doi:10.3389/fmed.2026.1752053)
Supplement: Supplementary file 1 [file Data_Sheet_1.zip › Supplementary material/Supplementary Table.docx]

**Table S1. Clinical characteristics of NPC patients treated with IMRT with or without CC in the original and matched cohorts**

| **Characteristic** | **The original cohort** | | ***P*-value** | **The matched cohort** | | ***P*-value** |
| --- | --- | --- | --- | --- | --- | --- |
|  | **IMRT** | **CCRT** |  | **IMRT** | **CCRT** |  |
|  | **(n = 524)** | **(n = 1510)** |  | **(n = 497)** | **(n = 497)** |  |
| **Gender** |  |  | 0.881 |  |  | 0.329 |
| Female | 143 (27.29%) | 407 (26.95%) |  | 138 (27.77%) | 152 (30.58%) |  |
| Male | 381 (72.71%) | 1103 (73.05%) |  | 359 (72.23%) | 345 (69.42%) |  |
| **Age** (years old) |  |  | < 0.001 |  |  | 0.52 |
| ≥45 | 313 (59.73%) | 719 (47.62%) |  | 286 (57.55%) | 296 (59.56%) |  |
| < 45 | 211 (40.27%) | 791 (52.38%) |  | 211 (42.45%) | 201 (40.44%) |  |
| **Smoking** |  |  | 0.042 |  |  | 0.675 |
| Yes | 154 (29.39%) | 517 (34.24%) |  | 148 (29.78%) | 142 (28.57%) |  |
| No | 370 (70.61%) | 993 (65.76%) |  | 349 (70.22%) | 355 (71.43%) |  |
| **Drinking** |  |  | 0.083 |  |  | 0.673 |
| Yes | 57 (10.88%) | 209 (13.84%) |  | 52 (10.46%) | 48 (9.66%) |  |
| No | 467 (89.12%) | 1301 (86.16%) |  | 445 (89.54%) | 449 (90.34%) |  |
| **Family history** |  |  | 0.282 |  |  | 1.000 |
| Yes | 159 (30.34%) | 421 (27.88%) |  | 154 (30.99%) | 154 (30.99%) |  |
| No | 365 (69.66%) | 1089 (72.12%) |  | 343 (69.01%) | 343 (69.01%) |  |
| **KPS** |  |  | 0.496 |  |  | 0.790 |
| ≥90 | 491 (93.70%) | 1472 (94.50%) |  | 466 (93.76%) | 468 (94.16%) |  |
| < 90 | 33 (6.30%) | 83 (5.50%) |  | 31 (6.24%) | 29 (5.84%) |  |
| **8th T stage** |  |  | < 0.001 |  |  | 0.347 |
| T1 | 121 (23.09%) | 252 (16.69%) |  | 115 (23.14%) | 129 (25.96%) |  |
| T2 | 174 (33.21%) | 322 (21.32%) |  | 153 (30.78%) | 134 (26.96%) |  |
| T3 | 229 (43.7%) | 936 (61.99%) |  | 229 (46.08%) | 234 (47.08%) |  |
| **8th N stage** |  |  | < 0.001 |  |  | 0.597 |
| N0 | 171 (32.63%) | 299 (19.8%) |  | 150 (30.18%) | 136 (27.36%) |  |
| N1 | 312 (59.54%) | 950 (62.91%) |  | 306 (61.57%) | 316 (63.58%) |  |
| N2 | 41 (7.82%) | 261 (17.28%) |  | 41 (8.25%) | 45 (9.05%) |  |

All continuous variables were changed to categorical variables. Pearson χ^2^ test was used to compute the *P*-value. CC, concurrent chemotherapy; CCRT, concurrent chemoradiotherapy; IMRT, intensity-modulated radiotherapy.

**Table S2. Clinical characteristics of NPC patients treated with IC followed by IMRT with or without CC in the original and matched cohorts**

| **Characteristic** | **The original cohort** | | ***P*-value** | **The matched cohort** | | ***P*-value** |
| --- | --- | --- | --- | --- | --- | --- |
|  | **IC + IMRT** | **IC + CCRT** |  | **IC + IMRT** | **IC + CCRT** |  |
|  | **(n = 216)** | **(n = 657)** |  | **(n = 209)** | **(n = 209)** |  |
| **Gender** |  |  | 0.582 |  |  | 0.588 |
| Female | 60 (27.78%) | 170 (25.88%) |  | 57 (27.27%) | 62 (29.67%) |  |
| Male | 156 (72.22%) | 487 (74.12%) |  | 152 (72.73%) | 147 (70.33%) |  |
| **Age** (years old) |  |  | 0.053 |  |  | 0.323 |
| ≥45 | 118 (54.63%) | 309 (47.03%) |  | 115 (55.02%) | 125 (59.81%) |  |
| < 45 | 98 (45.37%) | 348 (52.97%) |  | 94 (44.98%) | 84 (40.19%) |  |
| **Smoking** |  |  | 0.187 |  |  | 0.6 |
| Yes | 66 (30.56%) | 233 (35.46%) |  | 64 (30.62%) | 69 (33.01%) |  |
| No | 150 (69.44%) | 424 (64.54%) |  | 145 (69.38%) | 140 (66.99%) |  |
| **Drinking** |  |  | 0.025 |  |  | 1.000 |
| Yes | 19 (8.8%) | 97 (14.76%) |  | 19 (9.09%) | 19 (9.09%) |  |
| No | 197 (91.2%) | 560 (85.24%) |  | 190 (90.91%) | 190 (90.91%) |  |
| **Family history** |  |  | 0.366 |  |  | 0.371 |
| Yes | 63 (29.17%) | 171 (26.03%) |  | 58 (27.75%) | 50 (23.92%) |  |
| No | 153 (70.83%) | 486 (73.97%) |  | 151 (72.25%) | 159 (76.08%) |  |
| **KPS** |  |  | 0.893 |  |  | 0.674 |
| ≥90 | 205 (94.91%) | 622 (94.67%) |  | 198 (94.74%) | 196 (93.78%) |  |
| < 90 | 11 (5.09%) | 35 (5.33%) |  | 11 (5.26%) | 13 (6.22%) |  |
| **8th T stage** |  |  | 0.003 |  |  | 0.512 |
| T1 | 24 (11.11%) | 64 (9.74%) |  | 21 (10.05%) | 28 (13.4%) |  |
| T2 | 40 (18.52%) | 74 (11.26%) |  | 37 (17.7%) | 28 (13.4%) |  |
| T3 | 118 (54.63%) | 349 (53.12%) |  | 117 (55.98%) | 117 (55.98%) |  |
| T4 | 34 (15.74%) | 170 (25.88%) |  | 34 (16.27%) | 36 (17.22%) |  |
| **8th N stage** |  |  | 0.012 |  |  | 0.227 |
| N0 | 36 (16.67%) | 81 (12.33%) |  | 34 (16.27%) | 28 (13.4%) |  |
| N1 | 131 (60.65%) | 356 (54.19%) |  | 128 (61.24%) | 139 (66.51%) |  |
| N2 | 39 (18.06%) | 155 (23.59%) |  | 37 (17.8%) | 26 (12.44%) |  |
| N3 | 10 (4.62%) | 65 (9.89%) |  | 10 (4.78%) | 16 (7.66%) |  |

All continuous variables were changed to categorical variables. Pearson χ^2^ test was used to compute the *P*-value. CC, concurrent chemotherapy; CCRT, concurrent chemoradiotherapy; IC, induction chemotherapy; IMRT, intensity-modulated radiotherapy.
**Table S3. Clinical characteristics of NPC patients treated with CCRT with or without IC in the original and matched cohorts**

| **Characteristic** | **The original cohort** | | ***P*-value** | **The matched cohort** | | ***P*-value** |
| --- | --- | --- | --- | --- | --- | --- |
|  | **CCRT** | **IC + CCRT** |  | **CCRT** | **IC + CCRT** |  |
|  | **(n = 1176)** | **(n = 657)** |  | **(n = 533)** | **(n = 533)** |  |
| **Gender** |  |  | 0.295 |  |  | 0.382 |
| Female | 331 (28.15%) | 170 (25.88%) |  | 128 (24.02%) | 116 (21.76%) |  |
| Male | 845 (71.85%) | 487 (74.12%) |  | 405 (75.98%) | 417 (78.24%) |  |
| **Age** (years old) |  |  | 0.313 |  |  | 0.759 |
| ≥45 | 582 (49.49%) | 309 (47.03%) |  | 249 (46.72%) | 254 (47.65%) |  |
| < 45 | 594 (50.51%) | 348 (52.97%) |  | 284 (53.28%) | 279 (52.35%) |  |
| **Smoking** |  |  | 0.659 |  |  | 0.949 |
| Yes | 405 (34.44%) | 233 (35.46%) |  | 199 (37.34%) | 198 (37.15%) |  |
| No | 771 (65.56%) | 424 (64.54%) |  | 334 (62.66%) | 335 (62.85%) |  |
| **Drinking** |  |  | 0.704 |  |  | 0.441 |
| Yes | 166 (14.12%) | 97 (14.76%) |  | 76 (14.26%) | 85 (15.95%) |  |
| No | 1010 (85.88%) | 560 (85.24%) |  | 457 (85.74%) | 448 (84.05%) |  |
| **Family history** |  |  | 0.311 |  |  | 0.834 |
| Yes | 332 (28.23%) | 171 (26.03%) |  | 136 (25.52%) | 139 (26.08%) |  |
| No | 844 (71.77%) | 486 (73.97%) |  | 397 (74.48%) | 394 (73.92%) |  |
| **KPS** |  |  | 0.433 |  |  | 0.402 |
| ≥90 | 1103 (93.79%) | 622 (94.67%) |  | 503 (94.37%) | 509 (95.50%) |  |
| < 90 | 73 (6.21%) | 35 (5.33%) |  | 30 (5.63%) | 24 (4.50%) |  |
| **8th T stage** |  |  | < 0.001 |  |  | 0.389 |
| T1 | 203 (17.26%) | 64 (9.74%) |  | 53 (9.94%) | 60 (11.26%) |  |
| T2 | 259 (22.02%) | 74 (11.26%) |  | 83 (15.57%) | 68 (12.76%) |  |
| T3 | 614 (52.21%) | 349 (53.12%) |  | 309 (57.97%) | 303 (56.85%) |  |
| T4 | 100 (8.51%) | 170 (25.88%) |  | 88 (16.52%) | 102 (19.13%) |  |
| **8th N stage** |  |  | < 0.001 |  |  | 0.204 |
| N0 | 270 (22.96%) | 81 (12.33%) |  | 67 (12.57%) | 81 (15.2%) |  |
| N1 | 734 (62.41%) | 356 (54.19%) |  | 332 (62.29%) | 325 (60.98%) |  |
| N2 | 146 (12.41%) | 155 (23.59%) |  | 111 (20.83%) | 94 (17.64%) |  |
| N3 | 26 (2.22%) | 65 (9.89%) |  | 23 (4.31%) | 33 (6.18%) |  |

All continuous variables were changed to categorical variables. Pearson χ^2^ test was used to compute the *P*-value. CCRT, concurrent chemoradiotherapy; IC, induction chemotherapy.

**Table S4. Clinical characteristics of NPC patients treated with CCRT with or without AC in the original and matched cohorts**

| **Characteristic** | **The original cohort** | | ***P*-value** | **The matched cohort** | | ***P*-value** |
| --- | --- | --- | --- | --- | --- | --- |
|  | **CCRT** | **CCRT + AC** |  | **CCRT** | **CCRT + AC** |  |
|  | **(n = 1764)** | **(n = 51)** |  | **(n = 51)** | **(n = 51)** |  |
| **Gender** |  |  | 0.213 |  |  | 0.276 |
| Female | 485 (27.49%) | 10 (19.61%) |  | 6 (11.76%) | 10 (19.61%) |  |
| Male | 1279 (72.51%) | 41 (80.39%) |  | 45 (88.24%) | 41 (80.39%) |  |
| **Age** (years old) |  |  | 0.754 |  |  | 0.552 |
| ≥45 | 860 (48.75%) | 26 (50.98%) |  | 23 (45.1%) | 26 (50.98%) |  |
| < 45 | 904 (51.25%) | 25 (49.02%) |  | 28 (54.9%) | 25 (49.02%) |  |
| **Smoking** |  |  | 0.828 |  |  | 0.308 |
| Yes | 614 (34.81%) | 17 (33.33%) |  | 22 (43.14%) | 17 (33.33%) |  |
| No | 1150 (65.19%) | 34 (66.67%) |  | 29 (56.86%) | 34 (66.67%) |  |
| **Drinking** |  |  | 0.141 |  |  | 0.813 |
| Yes | 251 (14.23%) | 11 (21.57%) |  | 12 (23.53%) | 11 (21.57%) |  |
| No | 1513 (85.77%) | 40 (78.43%) |  | 39 (76.47%) | 40 (78.43%) |  |
| **Family history** |  |  | 0.523 |  |  | 0.664 |
| Yes | 482 (27.32%) | 16 (31.37%) |  | 14 (27.45%) | 16 (31.37%) |  |
| No | 1282 (72.68%) | 35 (68.63%) |  | 37 (72.55%) | 35 (68.63%) |  |
| **KPS** |  |  | 0.983 |  |  | 1.000 |
| ≥90 | 1659 (94.05%) | 48 (94.12%) |  | 48 (94.12%) | 48 (94.12%) |  |
| < 90 | 105 (5.95%) | 3 (5.88%) |  | 3 (5.88%) | 3 (5.88%) |  |
| **8th T stage** |  |  | 0.125 |  |  | 0.591 |
| T1 | 254 (14.4%) | 11 (21.57%) |  | 14 (27.45%) | 11 (21.57%) |  |
| T2 | 324 (18.37%) | 5 (9.8%) |  | 5 (9.8%) | 5 (9.8%) |  |
| T3 | 930 (52.72%) | 24 (47.06%) |  | 26 (50.98%) | 24 (47.06%) |  |
| T4 | 256 (14.51%) | 11 (21.57%) |  | 6 (11.77%) | 11 (21.57%) |  |
| **8th N stage** |  |  | 0.236 |  |  | 0.81 |
| N0 | 332 (18.82%) | 15 (29.41%) |  | 13 (25.49%) | 15 (29.41%) |  |
| N1 | 1053 (59.69%) | 28 (54.9%) |  | 31 (60.78%) | 28 (54.9%) |  |
| N2 | 292 (16.56%) | 7 (13.73%) |  | 5 (9.8%) | 7 (13.73%) |  |
| N3 | 87 (4.93%) | 1 (1.96%) |  | 2 (3.93%) | 1 (1.96%) |  |

All continuous variables were changed to categorical variables. Pearson χ^2^ test was used to compute the *P*-value. AC, adjuvant chemotherapy; CCRT, concurrent chemoradiotherapy.
