# Supplementary material for: Treatment and clinical outcomes among stage II–IVA nasopharyngeal carcinoma patients with undetectable pretreatment Epstein-Barr viral DNA
Source: Front Med (Lausanne). 2026 Aug 27;13:1752053. doi: 10.3389/fmed.2026.1752053 (PMC13558178; doi:10.3389/fmed.2026.1752053)
Supplement: Supplementary file 1 [file Data_Sheet_1.zip › Supplementary material/Supporting Materials.docx]

**Supporting Materials**

**The methodology of detecting plasma EBV DNA**

Before therapy, peripheral blood (5ml) was collected from each patient, placed in an

ethylene diamine tetra acetic (EDTA)-coated tube, and centrifuged at 1600 g for 15 min to isolate plasma and peripheral blood cells (PBC). DNA was extracted from plasma and PBC using the QIAamp Blood Kit (Qiagen, Hilden, Germany) and *blood and body fluid protocol* was used for DNA extraction per column and the final elution volume was 50 μl per column.

The concentration of EBV DNA in the plasma was measured using a real-time quantitative PCR assay targeting the BamH I-W region of the EBV genome. The sequences of the forward and reverse primers were: 5`-GCCAG AGGTA AGTGG ACTTT-3` and 5`-TACCA CCTCC TCTTC TTGCT-3` respectively. A dual fluorescently-labelled oligomer, 5`-(FAM) CACAC CCAGG CACAC ACTAC ACAT (TAMRA)-3` served as the probe. Sequence data for the EBV genome were obtained from the GenBank sequence database. Amplifications were performed in an Applied Biosystems 7700 Sequence Detector and then analyzed using the Sequence Detection System software (version 1.6.3) developed by Applied Biosystems (Foster City, CA).

The plasma EBV DNA concentration was calculated using the following equation: C = Q × (VDNA/VPCR)×(1/VEXT), in which C represents the target concentration in plasma (copies/ml), Q represents the target quantity (copy number) determined by PCR, VDNA represents the total volume of DNA obtained after extraction (typically 50 μl/Qiagen extraction), VPCR represents the volume of DNA solution used for PCR (typically 2 μl) and VEXT represents the volume of plasma extracted (typically 0.5 ml).

According to the testing kit adopted by our hospital, the sensitivity, including the lower limit of detection and the lower limit of quantification, is 400 copies/ml. The specificity, including interference resistance and cross-reactivity, also meets the national standards.
